# Bidirectional Microbiome-Gut-Brain-Axis Communication Influences Metabolic Switch-Associated Responses in the Mosquito *Anopheles culicifacies*

**DOI:** 10.3390/cells11111798

**Published:** 2022-05-31

**Authors:** Tanwee Das De, Punita Sharma, Sanjay Tevatiya, Charu Chauhan, Seena Kumari, Pooja Yadav, Deepak Singla, Vartika Srivastava, Jyoti Rani, Yasha Hasija, Kailash C. Pandey, Mayur Kajla, Rajnikant Dixit

**Affiliations:** 1 Laboratory of Host-Parasite Interaction Studies, ICMR-National Institute of Malaria Research, Sector-8, Dwarka, Delhi 110077, India; tanwee.das@gmail.com (T.D.D.); sharma.punita86@gmail.com (P.S.); sanjaycena51@gmail.com (S.T.); rcharu08@gmail.com (C.C.); cnayadav11@gmail.com (S.K.); yadavpuji60@gmail.com (P.Y.); deepak@pau.edu (D.S.); vartikasrivastava40@gmail.com (V.S.); yadavjyoti712@gmail.com (J.R.); pandey.kailash70@gmail.com (K.C.P.); kajla@uwalumni.com (M.K.); 2 Department of Biology, Indian Institute of Science Education and Research, Dr. Homi Bhabha Road, Pashan, Pune 411008, India; 3 School of Agricultural Biotechnology, Punjab Agricultural University, Ludhiana 141004, India; 4 Department of Biotechnology, Delhi Technological University, Shahbad Daulatpur, Main Bawana Road, Delhi 110042, India; yashahasija@gmail.com

**Keywords:** mosquito, blood-feeding, metabolic switch, gut-brain-axis communication, microbiome

## Abstract

The periodic ingestion of a protein-rich blood meal by adult female mosquitoes causes a drastic metabolic change in their innate physiological status, which is referred to as a ‘metabolic switch’. While understanding the neural circuits for host-seeking is modestly attended, how the gut ‘metabolic switch’ modulates brain functions, and resilience to physiological homeostasis, remains unexplored. Here, through a comparative brain RNA-Seq study, we demonstrate that the protein-rich diet induces the expression of brain transcripts related to mitochondrial function and energy metabolism, possibly causing a shift in the brain’s engagement to manage organismal homeostasis. A dynamic mRNA expression pattern of neuro-signaling and neuro-modulatory genes in both the gut and brain likely establishes an active gut–brain communication. The disruption of this communication through decapitation does not affect the modulation of the neuro-modulator receptor genes in the gut. In parallel, an unusual and paramount shift in the level of neurotransmitters (NTs), from the brain to the gut after blood feeding, further supports the idea of the gut’s ability to serve as a ‘second brain’. After blood-feeding, a moderate enrichment of the gut microbial population, and altered immunity in the gut of histamine receptor-silenced mosquitoes, provide initial evidence that the gut-microbiome plays a crucial role in gut–brain–axis communication. Finally, a comparative metagenomics evaluation of the gut microbiome highlighted that blood-feeding enriches the family members of the Morganellaceae and Pseudomonadaceae bacterial communities. The notable observation of a rapid proliferation of *Pseudomonas bacterial* sp. and tryptophan enrichment in the gut correlates with the suppression of appetite after blood-feeding. Additionally, altered NTs dynamics of naïve and aseptic mosquitoes provide further evidence that gut-endosymbionts are key modulators for the synthesis of major neuroactive molecules. Our data establish a new conceptual understanding of microbiome–gut–brain–axis communication in mosquitoes.

## 1. Introduction

The brain is a privileged organ in shaping an animal’s behavior from lower to higher taxa by guiding and managing the diverse nature of external and internal stimuli. While all the behaviors of any organism are finely orchestrated by multiple organs, it is the brain that directs and exchanges decision-making actions to regulate distinct organs’ functions. Unlike the human brain, which hosts billions of neurons, it is amazing to know how blood-feeding mosquitoes, having less than 100,000 neurons in their tiny brain, regulate diverse functions, such as finding a suitable source for sugar feeding, and blood-feeding, searching for a mate, and locating a proper oviposition site for egg-laying, etc. Decades of the research highlight that the molecular interaction of olfactory-derived odorant-binding proteins (OBPs), olfactory receptors (Ors), and environmental chemical cues (external cues) are central to shaping these behaviors [1,2]. Additionally, the innate physiological status of the mosquitoes, such as satiated/starved, mated/unmated, nutritional status, gravid, or not, accounts for the successful accomplishment of these behavioral activities [3]. Thus, how the miniature brain of mosquitoes harmonizes internal and external cues and affects decision-making abilities, is not yet known. Upon locating a suitable vertebrate host, a positive feeding decision stimulates the salivary glands to facilitate rapid blood meal ingestion by the adult female mosquitoes, and temporarily arrest the olfactory actions until 30 h of blood-feeding [1]. A recent study by Duvall et al. indicates that the activation of neuropeptide Y (NPY) signaling is essential in the suppression of host-seeking behavior for several days after blood feeding [4]. However, we have limited knowledge on how the mosquito’s brain regulates the binary behavioral switch responses (sugar to blood-feeding) and maintains organismal physiology in blood-fed females.

The fast engorgement of the mosquito’s gut with blood causes a drastic metabolic shift in the innate physiological status from sugar to a protein-rich diet, resulting in the alteration of cellular fuel sources. This ‘metabolic switch’ is expected to drive multiple organs’ engagement to perform their respective functions, such as osmoregulation by malpighian tubules, progressive blood meal digestion by the gut, nutrient mobilization, and activation of vitellogenesis in the fat body, and ovary development for egg maturation [5,6,7,8,9,10]. It is the central nervous system that supervises inter-organ communication impartially to manage inner physiological activities. Several neuromodulators, such as neuropeptides, neurotransmitters, and neurohormones, with a role in neuro-synaptic signal transmission and inter-organ communication, have been characterized in fruit flies [9,11]. However, a similar correlation between the gut metabolic switch and brain function modulation in mosquitoes is limited to the *Aedes aegypti*, where brain-secreted insulin-like-peptide 3 is reported to play a significant role in the regulation of blood meal digestion and egg development [12]. Only a few recent genetic studies have suggested the key role of a few neuropeptides e.g., neuropeptide-Y, short-neuropeptide F, and allatostatin-A, and their receptors in the suppression of host-seeking and paternity enforcement in *Aedes aegypti* mosquitoes [4,13,14]. Furthermore, it is becoming increasingly evident in vertebrates that an enteric nervous system (vagus nerve), also referred to as the ‘Second Brain’ [15] not only mediates cross-talk between the gut and the brain, but also establishes a bi-directional communication via gut-endosymbionts. This nexus of communication among the microbiota–gut–vagus–brain axis is crucial for maintaining metabolic homeostasis, mood, and perception [16,17,18]. Blood meal significantly modulates metabolic energy-related functions in the mosquito gut, but how the gut’s nutrient-sensing mechanism influences brain function remains unknown [19]. Although blood-meal-induced gut-flora proliferation has been well demonstrated in mosquitoes [20], their neuromodulatory functions remain elusive.

Using a comprehensive RNA-Seq analysis of mosquito brain, coupled with extensive transcriptional profiling of neuro-modulators, comparative metagenomics analysis, and LC/MS-based quantitative estimation of neurotransmitters, here we demonstrated that gut-metabolic switching (i) enhances the brain’s energy-metabolism, which may likely influence organismal homeostasis, and (ii) favor the rapid establishment of a bidirectional microbiome–gut–brain axis communication, where the gut may also serve as a secondary brain in the blood-fed mosquitoes. Our data suggest that this gut–brain–axis communication is crucial to guiding and managing the blood meal digestion, and vitellogenesis process in *Anopheles culicifacies* mosquitoes, the dominant malaria vector in rural India. A strategy of impairing this communication could reveal an out-of-the-box technique to disrupt mosquito host-seeking and blood-feeding behavior.

## 2. Material and Methods

A technical overview of the current investigation was represented graphically in Figure S1 (Supporting Information).

### 2.1. Mosquito Rearing and Maintenance

A cyclic colony of *An. culicifacies* mosquito, sibling species A was reared and maintained at 28 ± 2 °C temperature and relative humidity of 80% in the central insectary facility of the ICMR-National Institute of Malaria Research. For routine rearing, adult female mosquitoes were fed on the rabbit. All protocols for rearing and maintenance of the mosquito culture were approved by the ethical committee of the institute.

### 2.2. RNA Isolation and Transcriptome Sequencing Analysis

For RNA-Seq analysis, the brain tissues were dissected from 0–1-day old, 30 min post-blood-fed, and 30 h post-blood-fed cold anesthetized *An. culicifacies* mosquitoes by decapitation of the heads followed by gentle extraction of the brain with the use of sharp needle and forceps. During optimization of brain tissue dissection, we ensured that any eye pigment contamination could be prevented by careful handling of the dissecting needles and forceps. The dissected clean brain tissues (free of any eye pigment) were subsequently washed thrice in sterile nuclease free water (~20 μL) to avoid any fat-body and other surrounding tissue contamination, and pooled in Trizol reagent (RNAiso Plus, Cat. No. 9108, Takara, Japan). Total RNA was extracted from the collected clean brain tissues (approximately 30 mosquitoes were pooled to form one single sample), and a double-stranded cDNA library for each set of naïve, 30 min, and 30 h post-blood-fed was prepared by a prior well-established PCR-based protocol [21]. For transcriptome sequencing, the Illumina MiSeq 2× 150 paired-end library preparation protocol was followed. The accession numbers of the RNASeq sequencing data are SRR9853884 for Ac-Br-SF, SRR9853885 for Ac-Br-30 min, and SRR9853883 for Ac-Br-30 h. The bioinformatics data analysis pipeline is shown in Figure S1 and we followed a similar pipeline as discussed before. Briefly, raw reads from each set were processed to remove the adaptors and low-quality bases (<20). A de-novo clustering was used to build the final contigs/transcripts dataset using CLC Genomics Workbench (V6.2, Qiagen, Aarhus Denmark) with default parameters (contig length ≥ 200, Automatic word size: Yes, Perform Scaffolding: Yes, Mismatch cost: 2, Insertion cost: 3, Deletion cost: 3, length fraction: 0.5, Similarity fraction: 0.8). Finally, the assembled transcriptome was used for CDS prediction and annotation using transdecoder software and BLASTX at e-value 1e^−6^ (against a non-redundant database), respectively. For a comprehensive differential gene expression analysis, we used the same protocol as mentioned previously [1,21] and validated the DGE data by performing a quantitative real-time PCR with four different biological replicates (Table S1) Additionally, to identify the differentially expressed genes associated with certain biological and molecular processes, we performed pathway enrichment analysis using the Kobas 3.0 web server against PANTHER, KEGG Pathway databases. The unique appearance of certain pathways in different brain samples was screened depending on the *p*-value (<0.005).

### 2.3. PCR-Based Gene Expression Analysis

To establish the concept of the metabolic switch and inter-organ communication in mosquitoes, we targeted *An. culicifacies* brain, midgut, malpighian tubule, and ovary tissues. The respective tissues were dissected and collected from both naïve sugar-fed and blood-fed mosquitoes originated from the same cohort at different time points. At first, the tissues were collected from 5–6-days old 25–30 naïve sugar-fed adult female mosquitoes. Next, adult female mosquitoes from the same cohort were offered a blood meal by offering a live animal (rabbit), and the desired tissues were collected as per the technical design of the experiments. In general, the fully engorged females were separated and kept in proper insectary conditions and the tissues were collected at the selected time points of post-blood-meal (PBM) such as 5 min PBM, 2 h PBM, 8–10 h PBM, 24–30 h PBM, 48 h PBM, and 72 h PBM from 25–30 mosquitoes for tissue-specific detailed expression analysis of the respective genes. The different tissues were pooled accordingly in Trizol and total RNA was extracted, followed by cDNA preparation. Differential gene expression analysis was performed using the normal RT-PCR and agarose gel electrophoresis protocols. For relative gene expression analysis, SYBR Green qPCR master mix and Biorad CFX 96 Real-Time PCR machine were used. PCR cycle parameters involved an initial denaturation at 95 °C for 5 min, 40 cycles of 10 s at 95 °C, 15 s at 52 °C, and 22 s at 72 °C. Fluorescence readings were taken at 72 °C after each cycle. The final steps of PCR at 95 °C for 15 s followed by 55 °C for 15 s, and again 95 °C for 15 s were completed before deriving a melting curve. Each experiment was performed in three independent biological replicates for a better evaluation of the relative expression. The actin or Rps7 gene was used as an internal control in all the experiments, and the relative quantification was analyzed by the 2^−ΔΔCt^ method [22], which was further statistically analyzed by applying the student ‘*t*-test’ and two-way ANOVA. The detailed list of primer sequences used in the study is mentioned in Table S2.

### 2.4. ROS Determination Assay of Blood-Fed Mosquitos’ Brain

To unravel the origin of the oxidative stress response in the blood-fed brain, we performed a reactive oxygen species (ROS) determination assay by incubating the brain tissue dissected from naïve and blood-fed mosquitoes with a 2 mM solution of the oxidant-sensitive fluorophores, CM-H2DCFDA [5-(and-6)-chloromethyl-29,79-dichloro-dichlorofluorescein diacetate, acetyl ester] (Sigma, St. Louis, MO, USA). After a 20-min incubation at room temperature in the dark, the brain tissues were washed thrice with PBS, and then transferred to a glass slide in a drop of PBS and checked the fluorescence intensity at wavelength 490 nm under a fluorescent microscope.

### 2.5. Antibiotic Treatment of Mosquitoes

To establish the concept of microbiome–gut–brain axis communication, we disrupted the gut-commensal bacteria through antibiotic treatment. For the removal of gut bacteria, the pupae were kept in a washed and aseptic mosquito cage made up of muslin cloth for adult emergence. The antibiotic diet was provided to the newly emerged mosquitoes for 4–5 days by mixing 10% sucrose solution with 10 µg of penicillin-streptomycin/mL and 15 µg gentamicin sulfate in it. To avoid any contamination, the antibiotic regimen was changed daily. After 4–5 days of antibiotic treatment, rabbit was used to provide a blood meal to mosquitoes by maintaining proper sterile conditions such as (i) removing the extra hairs of rabbit pinnate/ears for easy access to blood meal, (ii) wiping the body of the rabbit with 70% ethanol, (iii) wiping the rabbit cage with 70% alcohol.

### 2.6. Decapitation Experiment

To test the mosquito’s gut ability to function as a second brain, we offered a blood meal to 5–6 days old naïve sugar-fed mosquitoes and decapitated ~100 mosquitoes after one hour of blood-feeding. Next, the decapitated mosquitoes were securely kept back in the insectary for recovery. The head tissues were submerged in 1× PBS to avoid desiccation. As per the technical design, post decapitation, the percentage of mosquito’s survival was recorded at different time points until we observed 100% mortality (mosquitoes that vibrate/move their legs or other body parts were considered as live and non-movable mosquitoes with visible shrinkage of the body parts at the respective time points were considered as dead). The brain and the gut tissues of surviving mosquitoes were dissected and collected at different time points for further gene expression analysis.

### 2.7. Sample Processing and MS Analysis for Neurotransmitter Quantification

For the absolute quantification of neurotransmitters, mosquitoes were decapitated and brains pulled out from the head cuticle and quickly collected in an Eppendorf containing 50 µL of 1% ascorbic acid and immediately frozen. For each set, ~60–65 mosquito brains or guts were pooled in a single tube. All samples were stored at −80 °C until further use. Each sample was extracted with 3× volume of extraction solvent. Samples were vortexed and refrigerated for 10–15 min at 4 °C. Samples were then subjected to sonication in a bath-type ultra-sonicator in pulses (twice, for 1 min each). Samples were then centrifuged at 14,500 rpm for 5 min at 4 °C. The supernatants were separated and dried under a vacuum. Dried samples were spiked with internal standards (ISTDs) and derivatized, cleaned up, and prepared for LC-MS injections as per the protocol described earlier [23].

Briefly, standards (STDs) were spiked in 200 μL of extraction solvent (acidic acetone (0.1% FA) containing 0.5 mM ascorbic acid) and dried under vacuum. ISTDs were spiked to dried STDs, followed by the addition of 80 μL borate buffer (200 mM, pH 8.8) containing 1 mM ascorbic acid. To the above mixture, 10 μL of 0.1 N NaOH was added, followed by the addition of AQC (from 1 mg mL^−1^ stock). Samples were incubated at 55 °C for 10 min. The reaction was stopped by the addition of 500 μL of acidic water (0.1% FA). The derivatized standards were cleaned-up using the RP-SPE cartridges using the previously optimized protocol [23,24]: activation with methanol, equilibration with water (0.1% FA), loading of samples, washing (twice) with water (0.1% FA), and elution with acetonitrile: methanol (80:20) containing 2% FA. The eluate was dried under vacuum and reconstituted in 50 μL of 0.5% acetonitrile. 10 μL of reconstituted standards were injected for UHPLC-MS/SRM analysis.

Data were acquired on a TSQ Vantage (triple stage quadrupole) mass spectrometer (Thermo Fisher Scientific, Waltham, MA, USA) coupled with an Agilent 1290 Infinity series UHPLC system (Agilent Technologies, Santa Clara, CA, USA). The UHPLC system was equipped with a column oven (set at 40 °C) and a thermo-controller for maintaining the auto-sampler at 10 °C. A C-18 column (2.1 mm × 100 mm, 1.8 μm, Agilent, Inc., Santa Clara, CA, USA) was used to perform the separation. The mobile phase solvent A was 10 mM ammonium acetate in water containing 0.1% formic acid, and solvent B was acetonitrile containing 0.1% formic acid. The gradient was optimized to get maximum separation (2% B at 0 min, 2% B at 3 min, 20% B at 20 min, 35% B at 25 min, 80% B at 25–27 min, 2% B at 27–35 min) at a flow rate of 200 μL min^−1^. The operating conditions were as follows: ionization mode: positive; spray voltage: 3700 V; capillary temperature: 270 °C; source temperature: 80 °C; sheath gas: 30 (arbitrary units); auxiliary gas: 10 (arbitrary units); collision gas: argon. Parent and product masses, S-lens voltages, and collision energies were used as per the previously optimized method [23,24]. Later we calculated the fold changes of all the neurotransmitters by considering naïve sugar-fed as control. Any NT that showed >1.00-fold change, indicated as an increase in concentration and those with <1-fold change indicated a decrease in concentration.

### 2.8. dsRNA-Mediated Gene Silencing

To silence the expression of Ac-Histamine-gated-chloride channel (HR), in-vitro transcription reaction was performed to prepare dsRNA by using a TranscriptAid T7 high-yield transcription kit (Thermo Fisher Scientific, Waltham, MA, USA). Purfied dsRNA was injected into cold anesthetized 1–2-day(s)-old *An. culicifacies* mosquitoes. Though, we performed four independent silencing experiments, we were able to secure the required number of survived mosquitoes from only two biological replicates. 3–4 days post dsRNA injection, midgut, carcass tissues were collected from 10 mosquitoes and pooled for RNA extraction and cDNA preparation. The silencing efficiency was evaluated through a quantitative real-time PCR. For the control group, age-matched mosquitoes were injected with LacZ dsRNA. To test the effect of HR silencing on gut–brain axis communication during the metabolic switch, a blood meal was provided to both the control and knockdown mosquitoes, and midgut tissues were collected 24 h after blood feeding.

### 2.9. Metagenomics Analysis & Microbiome Profiling

For the metagenomics study, we collected gut from 3–4 days old sugar-fed adult female mosquitoes (*n* = 50). While for blood-fed mosquito gut samples, 3–4 days old adult female mosquitoes from the same cohort were provided a blood meal by offering a live animal (rabbit), and midguts were collected after 24–30 h of blood-feeding. Before dissection, the mosquitoes were surface sterilized with 70% ethanol for 1 min in the highly sterilized condition of the laminar airflow. At least 50 whole guts either from naïve sugar-fed or blood-fed mosquitoes, originating from the same cohort were collected into the minimal volume (20 µL) of sterile ice-cold 1× Saline Tris-EDTA (100 mM NaCl/10 mM Tris-HCl, pH 8.0/1 mM EDTA, pH 8.0) buffer, and whole DNA was extracted as described earlier [25]. The quality of extracted genomic DNA (gDNA) was checked by loading the 5 μL aliquot on 1% agarose gel under the condition of 110 V for 30 min. 1 µL of each sample was loaded in NanoDrop 8000 for determining the A_260/280_ ratio. The DNA was quantified using the Qubit dsDNA BR Assay kit (Thermo Fisher Scientific, Waltham, MA, USA). Moreover, 1 µL of each sample was used for determining concentration using a Qubit^®^ 2.0 Fluorometer. For the preparation of amplicon libraries, V3-V4 hyper-variable region primers were used according to the library preparation protocol for the 16S Metagenomic Sequencing. The library for the sequenced fragments was obtained as per the standard Illumina protocol. After trimming, a quality check was performed using FASTQC to ensure a score over 20 for all the bases. These trimmed sequences were then classified using taxonomic classifier kraken 2 through an automated pipeline workflow module available in the licensed software Omicsbox [26]. The accession numbers of the metagenomics sequencing data are SRR12579422 for Ac-MG-SF, and SRR12622557for Ac-MG-BF. To validate the metagenomics data, the abundance of the selected bacterial species was profiled through a real-time PCR, as described earlier [25].

## 3. Results

Since the neuro-system is a highly sensitive and versatile center for chemical information exchange, we hypothesize that a minor change in the innate physiological status may have a strong impact on the mosquito’s everyday life. For example, blood meal ingestion causes a drastic change in the gut metabolic machinery. Therefore, it is plausible to propose that fast engorgement of mosquito gut with blood meal may shift mosquitoes’ brain functions from external communication to inter-organ management, such as (a) initiation of diuresis; (b) digestion of blood meal in the midgut; (c) distribution of amino acids, generated through the degradation of protein-rich blood meal; (d) active engagement of the fat body and ovary for egg maturation and life cycle maintenance. Our previous study demonstrated that blood-meal ingestion suppresses olfactory responses for 30 h of blood-feeding until the blood is digested in the midgut of the female *An. culicifacies* mosquito [1,27,28] Aligning to olfactory responses, here, we aimed to decode a possible molecular correlation between the brain and gut-metabolic switch, and designed a similar RNA-Seq strategy, in the mosquito *An. culicifacies*.

### 3.1. Blood Meal Ingestion Enhances the Brain’s Energy Metabolism

**Figure 1:**
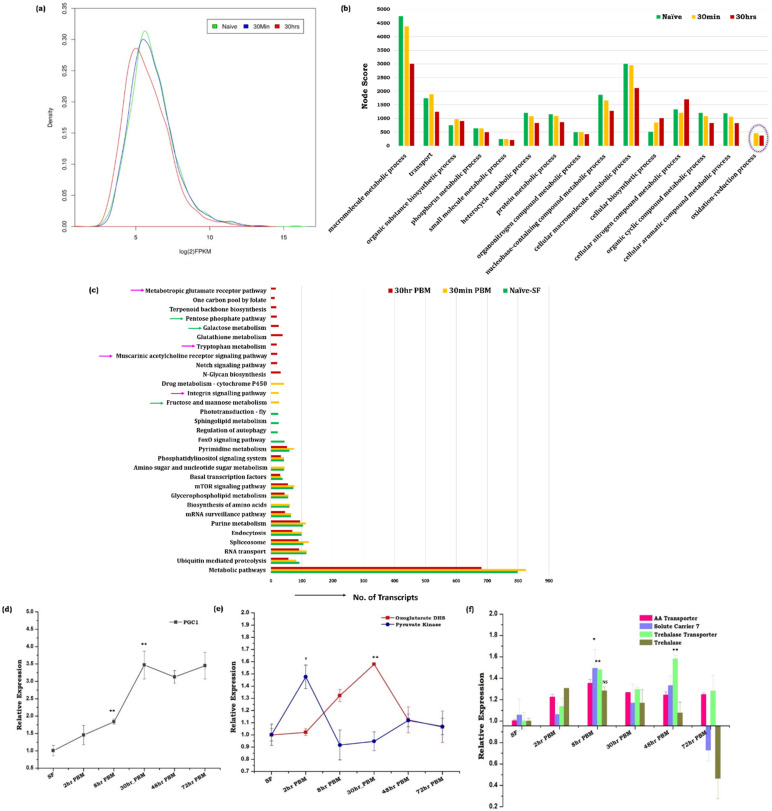
Blood meal causes notable changes in the molecular architecture of the brain tissue. (**a**) Comparison of the read density map of the naïve, 30 min, and 30 h post blood meal (PBM) transcriptomic data of brain tissue (*n* = 25); (**b**) Functional annotation and molecular cataloging of brain transcriptome (Biological Process/Level4/Node score). Purple circle highlighted the unique category of genes that appeared in the brain tissue after blood meal intake; (**c**) KOBAS 3.0 software mediated gene list enrichment and comparative pathway analysis of naïve and blood-fed brain tissues. Green arrow links to energy metabolic pathways, the pink arrow links to neurotransmitter synthesis and synaptic signaling pathway; (**d**) Relative expression profiling of PGC-1 gene in the brain of naïve and blood-fed mosquitoes (*n* = 25, *N* = 3) (*p* ≤ 0.009 at 8 h PBM, *p* ≤ 0.007 at 30 h PBM) (*p* ≤ 0.005 is indicated as ‘**’)*;* (**e**) Transcriptional profiling of transcripts related to energy metabolism in the brain tissue of naïve and blood-fed mosquitoes at different time points. For pyruvate kinase the *p* value is ≤0.0176 at 2 h PBM, and oxo-glutarate dehydrogenase the *p* value is *p* ≤ 0.0019 at 30 h PBM (*p* ≤ 0.005 is indicated as ‘**’, *p* ≤ 0.05 is indicated as ‘*’); (**f**) Comparative transcriptional response of amino acid transporters and trehalose transporter along with trehalase enzyme in the brain tissue after the metabolic switch (*n* = 25, *N* = 3). For solute carrier 7 the *p* value is ≤0.0515 and for trehalose transporter the *p* value is (*p* ≤ 0.0071) Statistically significant variation in the expression of the respective genes was tested by the *t*-test and compared with the sugar-fed control brain. (*n* = number of mosquitoes from, which the respective tissue was dissected and pooled for each independent experiment; *N* = number of biological replicates). SF = naïve sugar-fed, 2 h-PBM (Post-Blood-Meal); 8 h-PBM; 30 h-PBM; 48 h-PBM; 72 h-PBM.

A read-density map and comparative gene ontology analysis of naïve sugar-fed, 30 min, and 30 h post-blood-fed RNA-Seq data of mosquito’s brain showed a gradual suppression of brain-specific transcript abundance (Figure 1a,b and Table S3a,b). Simultaneously, we also observed an exceptional enrichment of oxidation-reduction process associated transcripts including several mitochondrial activity proteins such as 2-oxoglutarate dehydrogenase, NADH dehydrogenase, glutathione peroxidase, etc., in response to blood-feeding (Figure 1b and Table S3a,b). However, we failed to detect any signal of oxidative stress in a 2 mM solution of the oxidant-sensitive fluorophores, CM-H2DCFDA (data are not shown). A comparative metabolic pathway prediction analysis further confirmed the exclusive induction of several unique pathways linked to (a) energy metabolism, (b) neurotransmitter synthesis, and (c) synaptic transmission (Figure 1c). Together, these data indicated that blood meal-associated gut metabolic switch may trigger the brain’s energy metabolism, and influence the expression of neuro-modulatory factors in the mosquito brain.

To verify the above presumption, we profiled and compared the expression pattern of the PGC-1 gene (peroxisome proliferator-activated receptor gamma coactivator 1-alpha), an important transcriptional co-activator that regulates genes involved in energy metabolism [29,30,31]. A persistent elevation of PGC-1, and a parallel enrichment of glycolysis and TCA cycle gene pyruvate kinase and oxoglutarate dehydrogenase respectively, indicated an enhanced mitochondrial activity in the brain of blood-fed mosquitoes (Figure 1d,e). Next, we tested whether the amino acids generated through blood meal digestion or trehalose, a non-reducing disaccharide, act as raw material for the brain’s energy metabolism. Although trehalose serves as a primary energy source in the insects’ brains [32,33], we observed a sequential increment in the amino acid transporter (solute carrier 7), as well as trehalose transporter genes in the blood-fed brain (Figure 1f). Together, these data indicate that both amino acids and trehalose moieties may likely work synergistically to communicate the nutritional signal to the brain for the active management of multi-organ communication [12,34].

### 3.2. Spatial and Temporal Modulations of Neuro-Signaling Influence Metabolic Switch-Associated Physiological Activities

In naive sugar-fed mosquitoes, external stimuli guided neuro-signaling and the brain’s energy consumption is balanced to drive routine behavioral events like flight, mating, and host-seeking. However, an increase in the brain’s energy metabolism following blood-feeding prompted us to test the functional correlation of the brain with gut metabolic switch activities. Here, we hypothesize that blood meal uptake may temporarily pause the external communication, and an increased energy state possibly may favor the shifting of the brain’s engagement for the maintenance of organismal homeostasis. Thus, we identified and shortlisted transcripts encoding proteins, likely involved in the key events of the synaptic signal transmission process including neurotransmitter receptors and cellular signaling molecules, and evaluated their blood-meal-associated transcriptional responses.

**Figure 2:**
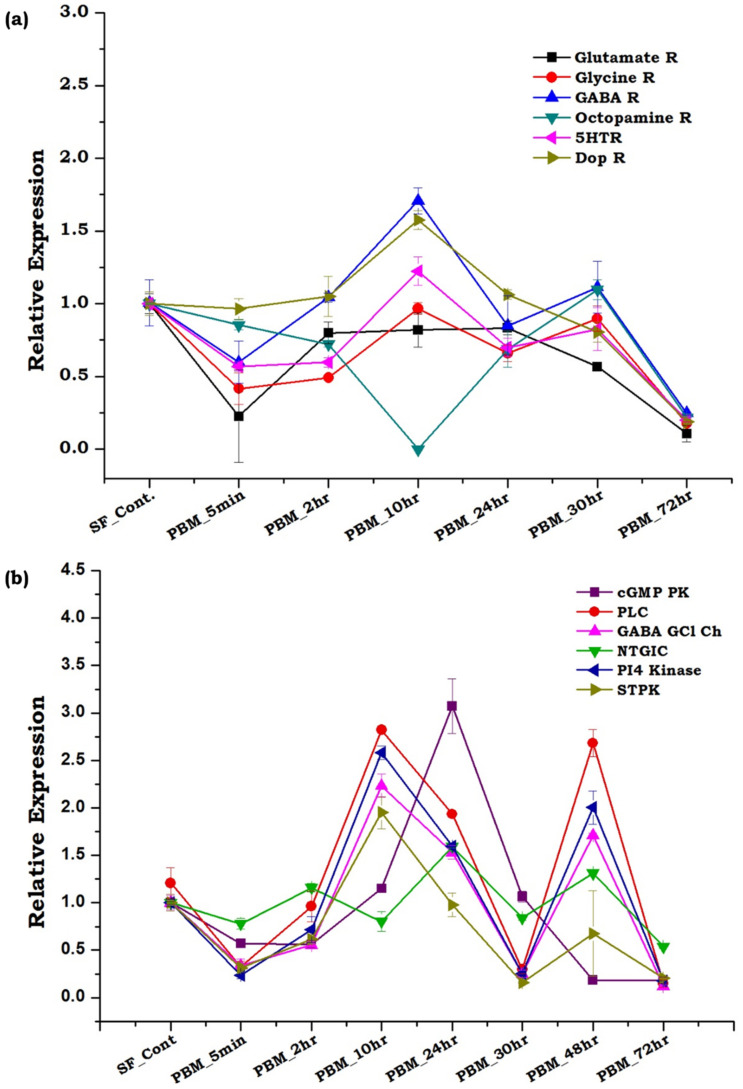
Metabolic switch influences neuro-signaling modulation and inter-organ communication. (**a**) Transcriptional response of neurotransmitter receptor genes as per the designed blood meal time-series experiment. Brain tissues were collected from 5–6 day old naïve sugar-fed adult female mosquitoes. Then, mosquitoes were provided with blood meal, and the brain tissues were collected at different time points after blood feeding viz. 5 min post blood meal (PBM-5 min), PBM-2 h, PBM-10 h, PBM-24 h, PBM 30 h, and PBM-72 h. Glutamate R: Glutamate Receptor; Glycine R: Glycine Receptor; GABA R: Gamma-Aminobutyric Acid Receptor; Octopamine R: Octopamine Receptor; 5 HTR: Serotonin Receptor; Dop R: Dopamine Receptor. Statistical analysis using two-way ANOVA has implied at 0.05 level the expression pattern of the respective genes was not statistically significant at *p* ≤ 0.2 at different time points after blood feeding (*n* = 25, *N* = 4); (**b**) Relative expression profiling of the genes involved in signal transduction molecules according to the detailed blood meal time-series experiment. cGMP PK: Cyclic GMP Protein Kinase; PLC: Phospholipase C; GABA GClCh: GABA Gated Chloride Channel; NTGIC: Neurotransmitter Gated Ion Channel; PI4 Kinase: Phosphatidyl-inositol-4-Kinase; STPK: Serine Threonine Protein Kinase. Statistical analysis using two-way ANOVA and Tukey’s test stated that the expression change of the respective genes is statistically significant *p* ≤ 0.05 (*n* = 25, *N* = 4). For Statistical analysis, all the time points and all the transcripts are compared together using two-way ANOVA and the means of the time points are statistically significant. (*n* = number of mosquitoes from which the respective tissue was dissected and pooled for each independent experiment; *N* = number of biological replicates).

Surprisingly, we observed no significant difference in the expression of neurotransmitters and biogenic amine receptor genes such as serotonin receptor, dopamine receptor, octopamine receptor GABA receptor, etc. in response to blood-meal (Figure 2a). While, on the contrary, cellular signal transduction proteins such as cGMP protein kinase, phospholipase C, GABA gated chloride channel, and serine-threonine protein kinase, exhibited a significant modulation in response to metabolic switch (Figure 2b). In parallel, to ensure the effectiveness of the blood-meal on neuro-signaling modulation in the aging mosquitoes, we monitored the expression profile of at least 14 neuro-modulatory genes in aging naïve sugar-fed adult female mosquitoes’ brains (Figure S2 and Table 1). We did not observe any significant changes in the expression of the transcripts in naïve sugar-fed mosquitoes of varied age groups. Together, these findings support the idea that a rapid blood meal ingestion may drive brain engagement to manage metabolic switch-associated activities and distant organs’ function.

### 3.3. Innate Physiological Status Differentially Modulates Tissue-Specific Neuromodulators/Receptors Transcripts Expression

**Figure 3:**
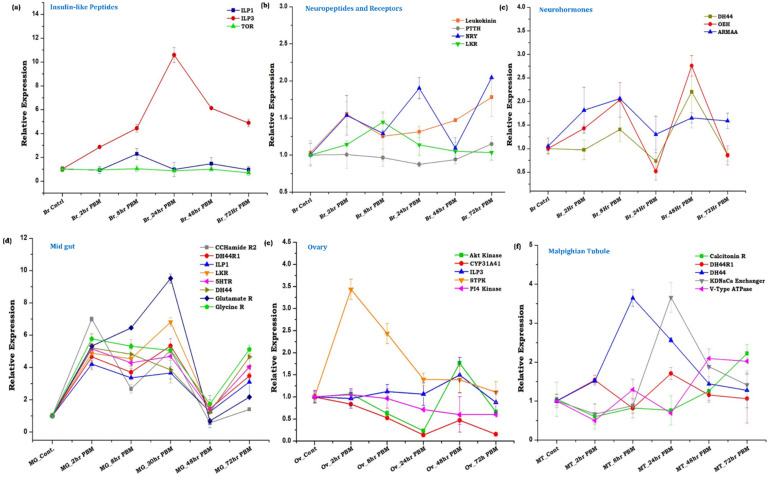
Metabolic switch modulates tissue-specific neuro-modulator transcripts expression. (**a**–**c**) Transcriptional expression profiling of Insulin-like-peptides, neuropeptides, neurohormones, and receptor genes in the brain tissue during the metabolic switch. Statistical analysis using two-way ANOVA and Tukey’s test implied that the expression change of the respective genes is statistically significant for insulin-like-peptides *p* ≤ 0.007; neuropeptides and receptors *p* ≤ 0.009, but for neuro-hormones, it was non-significant *p* ≤ 0.2 (*n* = 25, *N* = 4); (**d**) Relative expression profiling of a subset of neuromodulator genes in the midgut of naïve and blood-fed mosquitoes at the same time point described above. Statistical analysis using two-way ANOVA implied that the expression change of the respective genes is statistically significant *p* ≤ 0.005 (*n* = 12, *N* = 4)*;* (**e**) Transcriptional profiling of genes involved in signal transduction during vitellogenesis in the ovary. Statistical analysis using two-way ANOVA and Tukey’s test indicated that the expression change of the respective genes was statistically significant at *p* ≤ 0.002 (*n* = 12, *N* = 4); (**f**) Relative gene expression analysis of diuresis-related genes in the Malpighian tubule of naïve and blood-fed mosquitoes. Statistical analysis using two-way ANOVA and Tukey’s test indicates that the expression change of the respective genes is non-significant at *p* ≤ 0.4 (*n* = 25, *N* = 4). For Statistical analysis, all the time points and all the transcripts are compared together using two-way ANOVA and the means of the time points showed statistically significant. (*n* = number of mosquitoes from which the respective tissue was dissected and pooled for each independent experiment; *N* = number of biological replicates).

To further validate and correlate brain–distant-organ communication, we monitored the temporal and spatial expression of at least 21 key genes (Table 1) with a blood-meal-associated function in their targeted tissue, such as midgut (MG), ovary (Ov), and malpighian tubules (MT). Notably, we observed a significant upregulation of ILP3 (*p* < 0.0002), and the time-dependent modulation of other neuropeptides (Neuropeptide Y, Leukokinin) and neuro-hormones (OEH, DH44, and ARMAA) transcripts in the blood-fed mosquitoes’ brain (Figure 3a–c). We correlate that a gradual induction of ILP3 and OEH transcripts expression may replenish the stored peptides in the neurosecretory cells, which upon activation following blood-meal, secrete ILP3 and OEH to stimulate ecdysteroids synthesis from the ovaries [48,49,50]. A transient increase in NRY transcript immediately after blood-feeding may be due to gut distension, but a significant increase (*p* < 0.005) after 24 h and 72 h may cause suppression of host-seeking, a mechanism recently reported in *Aedes aegypti* [4,38].

Next, we asked how the dynamic changes of the neuromodulators in the blood-fed brain influence distant organ responses, such as diuresis regulation by the Malpighian tubule, blood digestion process in the midgut, and oocyte maturation in the ovary. Transcriptional profiling of selected neuropeptide, and neurotransmitter receptor transcripts (Table 1) indicated that blood meal triggers an immediate and prolonged (~48 h PBM) impact on the expression of the gut-neuro transcript (Figure 3d). Parallel observation of an early induction (2 h PBM) of serine threonine-protein kinase (MAPK activated protein kinase) and late expression of Akt kinase (48 h PBM) in the ovary also indicates the restoration of stored peptides which get activated through phosphorylation for the regulation of the vitellogenesis process (Figure 3e) [8,46]. Likewise, the observation of a unique pattern of diuretic hormone (8 h PBM) and potassium-dependent sodium-calcium exchanger gene (24 h PBF) expression in the malpighian tubule suggested an active diuresis process until 24 h post blood meal (Figure 3f) [40].

### 3.4. Gut, as the ‘Second Brain’ Communicates the Nutritional Status through Neurotransmitter Synthesis

In the vertebrates and in fruit flies, it is evident that the effective communication between the gut and brain has a paramount effect in shaping optimal health [15,17], but very limited knowledge exists in the mosquitoes [12]. The prolonged modulation of the neuromodulators’ gene expression in the blood-fed mosquitoes’ gut invigorates us to presume the existence of bi-directional gut–brain–axis communication. An enriched expression pattern of neurotransmitter receptor genes, even after decapitation, reflected that the gut may also perform neuro-modulatory actions independently (Figure S3). To further establish a proof-of-concept, we followed the LC/MS-based absolute quantification of different neurotransmitters (NT), and compared their levels in the brain as well as in the gut of naïve and blood-fed mosquitoes.

**Figure 4:**
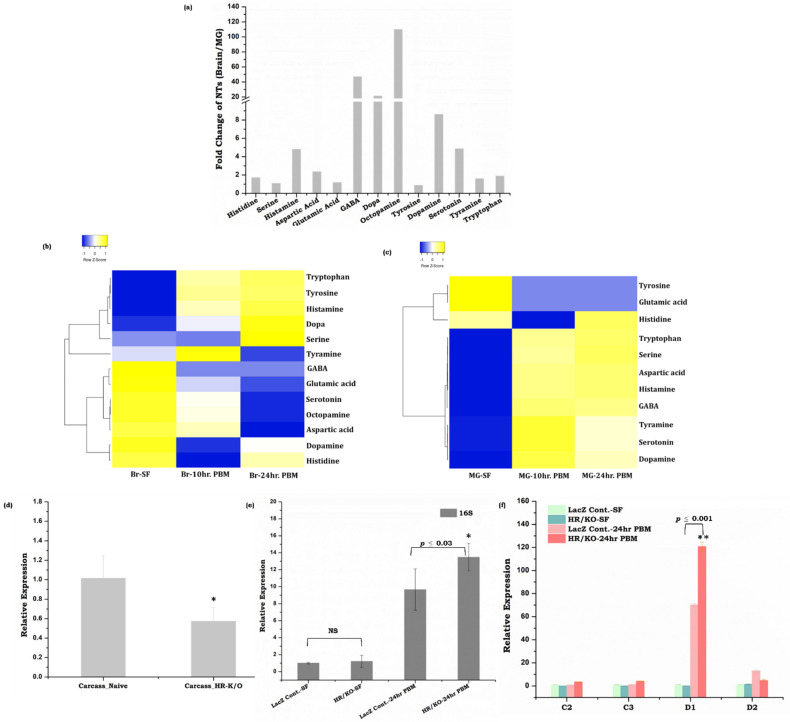
Gut–Brain–Axis (GBA) communication and neurotransmitter (NT) estimation in mosquito *An. culicifacies*. (**a**) Comparative analysis of NT abundance in the naïve mosquitoes’ brain and midgut; (**b**) Heatmap showing the alteration of neurotransmitters level in mosquito brain tissue. NT levels were measured by LC-MS from the brains of naïve (sugar-fed) and blood-fed females (10 and 24 h PBM) (*n* = 65, *N* = 2). Statistically significant differences in the amount of metabolites were tested by *p*-values (*p* ≤ 0.005) that are deduced by two-way ANOVA and Tukey’s test; (**c**) Heatmap of neurotransmitters levels of mosquito gut tissue that vary during the metabolic switch. NT levels were measured by LC-MS from the gut of naïve (sugar-fed) and blood-fed females (10 and 24 h PBM) (*n* = 50, *N* = 2). Statistically significant differences in the amount of metabolites were tested by *p*-values (*p* ≤ 0.005) that are deduced by two-way ANOVA and Tukey’s test. (*n* = number of mosquitoes from which the respective tissue was dissected and pooled for each independent experiment; *N* = number of biological replicates); (**d**) AcHR silencing validation in the carcass of *An. culicifacies* mosquitoes (*n* = 10, *N* = 2) (*p* ≤ 0.01) (*p* ≤ 0.05 is indicated as ‘*’). (**e**) Relative expression of 16S transcripts in control and HR knockdown mosquitoes. (**f**) Transcriptional response of immune genes in control vs HR knockdown mosquitoes during naïve sugar-fed and blood-fed conditions. *p* ≤ 0.005 is indicated as ‘**’, *p* ≤ 0.05 is indicated as ‘*’. C1: Cecropin 1, C2: Cecropin 2, D1: Defensin 1, D2: Defensin 2. Statistically significant differences for the silencing experiments are deduced by Students’ *t*-Test.

Our data revealed that in naïve sugar-fed mosquitoes, the brain contains higher levels of all the NTs compared to the gut (Figure 4a). However, blood-feeding caused a drastic shift in the NTs level in the midgut than in the brain (Figure 4b,c). Notably, we observed a 4–100-fold increase in most NTs except glutamic acid and tyrosine in the gut (Figure 4c and Figure S4a). Whereas, the brain tissue showed a notable decrease in the majority of the NTs synthesis (<0.6-fold decrease in eight NTs) and >1–4-fold increase in histamine, tyrosine, Dopa, and tryptophan (Figure 4b and Figure S4b). We also observed that tyrosine amino acid was exclusively induced in the brain after blood-feeding, but remained below the threshold level in the gut (Figure 4b,c and Figure S4a,b). Although our data support previous studies that along with the brain, the gut also serves as a major source of multiple neurotransmitters in vertebrates and fruit flies [15,51], the mechanism of nutrition-dependent NTs modulation remains unclear. Especially, in mosquitoes our understanding of the complex nature of blood meal digestion and gut-brain axis communication is obscure. Thus, our unusual observation of a hundred-fold increase in the levels of histidine, serine, aspartic acid, and tryptophan in the blood-fed mosquito’s gut emanated a few key questions: (1) whether increased levels of amino acids in the gut during blood meal digestion may act as an NT? (2) Do blood-meal-induced proliferation of the gut microbiota has any effect on NT dynamics? (3) Do the gut endosymbionts of mosquitoes have any impact on gut–brain axis communication? (Figure S4c).

To answer these questions and to correlate neurotransmitter abundance with endosymbionts, we opted to prevent histamine (one of the most abundant NT following blood-meal) signaling by knocking down the histamine-gated-chloride channel (HR) gene. Our dsRNA-mediated knock-down study indicated that disruption of histamine signaling not only influences microbial proliferation following blood-feeding, simultaneously it also modulates gut immune response (Figure 4d–f), though further studies are needed to explore the detailed mechanism of neurotransmitters and endosymbionts mediated gut–brain–axis communication.

### 3.5. Symbiotic Gut Flora Influences Gut-Brain Axis Communication

The mechanism of gut–brain axis communication in vertebrates primarily involves neuronal stimulation through the vagus nerve, where endosymbionts play a key role in the regulation of the gut endocrine system, and associated biochemical pathways [17,18]. The previous literature suggests that mosquito gut endosymbionts regulate many biological functions such as mosquito immunity, blood meal digestion, and ecological adaptation [42,52]. The ingestion of the protein-rich blood meal favors the rapid enrichment of gut microbiota [20], but whether it affects the nexus of communication between the gut and brain remains elusive.

**Figure 5:**
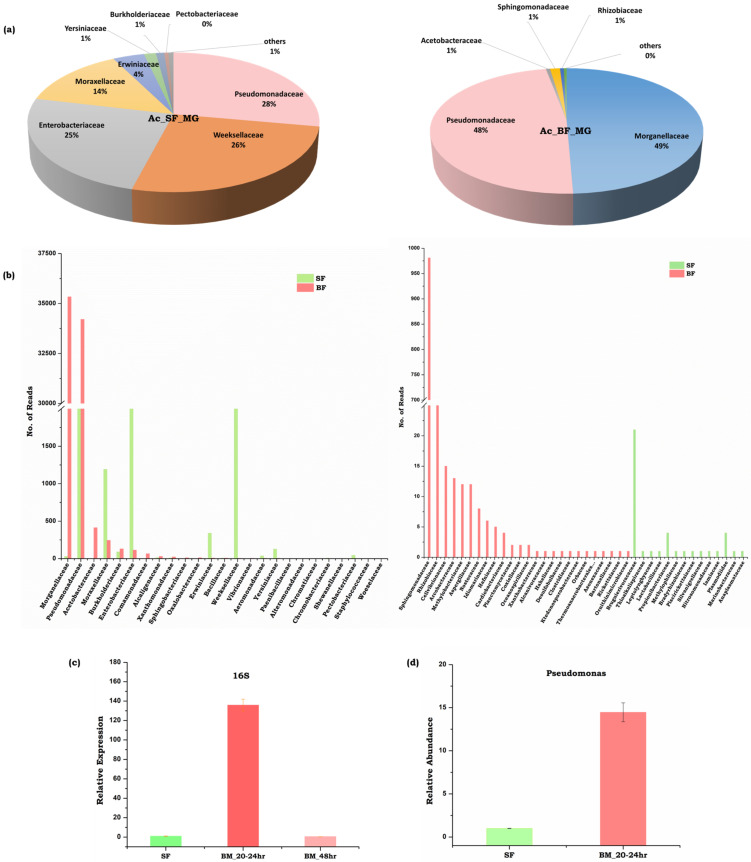
Comparison of gut-metagenomes in the naïve sugar-fed and blood-fed mosquito *Anopheles culicifacies*: (**a**) Pie charts representing the major bacterial families under the two feeding status (**b**) Number of reads based comparative bar graphs showing common and unique families microbes (**c**) Relative quantitative distribution of microbiota based on 16SrRNA based expression in the midgut of *An*. *culicifacies* in response to sugar and post blood feeding (20–24 h PBM, 48 h PBM); (**d**) Relative abundance of *Pseudomonas* sp*. bacteria* in sugar-fed and blood-fed (20–24 h PBM) condition.

Therefore, to uncover the gut microbiome diversity, and establish their possible relations with neurotransmitter abundance, we evaluated the population dynamics of the gut microbiome in response to blood-feeding. A comparative metagenomic analysis (Table S4) revealed that the naïve sugar-fed mosquitoes harbor 25% Enterobacteriaceae family of gram-negative gamma-proteobacteria such as *Klebsiella pneumonia*, *Salmonella enterica*, *E. coli*, *Tatumella ptyseos*; 28% Psedomonodaceae family of gram-negative gamma-proteobacteria, such as (a) *Acinetobacter* sp. members e.g., *Acinetobacterbaumannii*, *Acinetobacter seifertii*, (b) *Pseudomonas* sp. group e.g., *Pseudomonas putida*, *P.* sp. *SC3*, *P. aeruginosa* and *P. monteilii*; and other bacterial family members of Bacteroidetes e.g., Flavobacteriacae—*Chryseobacterium* sp., (Figure 5a,b). Furthermore, we also observed that blood-feeding not only suppressed Enterobacteriaceae family members, but favored the rapid proliferation of Pseudomonadales to more than 40% of the total community, where we observed the dominant association of *Pesudomonas mosselii*, and other members from the Alpha-proteobacteria family, such as *Asaia bogorensis* as well as Gamma-proteobacteria-Morganellaceae family members such as *Morganella morganii*. Our microbial profiling data further suggested that the blood meal significantly alters the abundance of the Gram-negative bacteria such as *Pseudomonas* sp. e.g., *P. mosselii*, *P. aeruginosa*, *P. chlororaphis* and *P. monteilii* (Figure 5)*,* and decreased the population of *E. coli*, *Salmonella* and *Klebsiella pneumonia* (Figure S5).

Although establishing the correlation between the abundance of Enterobacteriaceae family members’ and the low NTs level in the gut of sugar-fed mosquitoes needs further studies, our preliminary LC/MS data indicate that the basal level of gut–brain–axis communication may be enough to maintain the physiological homeostasis in naïve mosquitoes. However, there was a rapid proliferation of Pseudomonadales family members, and the multi-fold enrichment of NTs in the gut, while the mild suppression (0.9–0.5-fold) of the majority of NTs in the brain of the blood-fed mosquitoes suggests that members of the Pseudomonas species, may likely play a neuro-modulatory role in protein-rich diet-induced gut–brain–axis communication.

**Figure 6:**
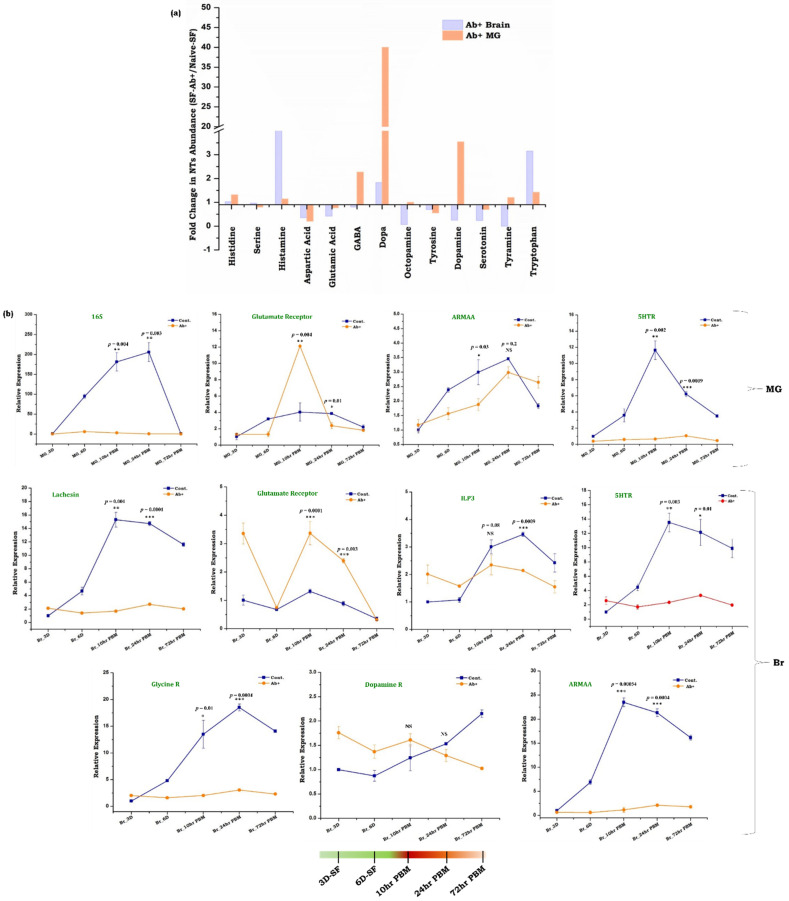
Establishing Microbiome–Gut–Brain–Axis (MGB) communication in mosquitoes. (**a**) Absolute quantification of the neurotransmitters (NT) in the brain and gut tissues of naïve sugar-fed and antibiotic-treated mosquitoes (*n* = 65, *N* = 2) which are represented as fold-change of NT abundance when compared to naïve sugar-fed conditions. Statistically significant differences in the amount of metabolites were tested by *p*-values (*p* ≤ 0.005) that are deduced by two-way ANOVA and Tukey’s test, (*n* = 50, *N* = 2); (**b**) Relative expression profiling of the 16S gene to show the population of microbial flora and other neuro-transcripts in the gut and brain of naïve and antibiotic-treated mosquitoes undergoing metabolic switch. Statistical significance of differences of the respective genes in control (without antibiotic) and aseptic mosquitoes (antibiotic-treated) were tested by the *t*-test. *p* ≤ 0.0005 is indicated as ‘***’, *p* ≤ 0.005 is indicated as ‘**’, *p* ≤ 0.05 is indicated as ‘*’. (*n* = number of mosquitoes from which the respective tissue was dissected and pooled for each independent experiment; *N* = number of biological replicates).

To further strengthen our hypothesis, we evaluated the effect of gut flora removal on the neurotransmitter’s dynamics. We performed an absolute quantification of the potent neuroactive molecules and compared their levels in the gut and brain of the naïve and antibiotic-treated mosquitoes. Moreover, 3-fold and 1.4-fold enhancements of tryptophan abundance and consequent 0.2-fold and 0.7-fold decreases in the amount of serotonin levels in both the brain and gut of aseptic non-blood fed mosquitoes (Figure 6a), corroborate with the previous observations that depletion of microbial flora may significantly delimit the de novo synthesis of serotonin, resulting in increased tryptophan concentration [53]. Additionally, we also observed that antibiotic treatment causes a notable increase in histidine and histamine levels in both the gut (1.3-fold and 1.14-fold respectively) and brain (1.02-fold and 4.8-fold respectively) (Figure 6a,b). An exclusive induction of Dopa (40-fold), and significant enrichment of GABA (2.3-fold) were also noticed in the gut of the aseptic mosquitoes (Figure 6a). Though the correlation of the microbiome–gut–brain axis communication in the blood-fed mosquitoes is not yet fully established, together, these data indicated that amino-acids resulting from the rapid digestion of protein-rich blood meal, and its metabolite products may serve as an additional potent source of neuromodulators (Figure 6a,b).

To understand the effect of antibiotics on metabolic switch-induced gut–brain axis communication, we quantified and compared the level of the neurotransmitters of naïve and antibiotic-treated blood-fed mosquitoes. A similar pattern of NTs synthesis was observed in both naïve and antibiotic-treated blood-fed mosquitoes, but the level of modulation is heightened in the antibiotic-treated blood-fed gut and brain (Figure S6 and Table S5). To further support the above observation, we also monitored and compared the expression patterns of neurotransmitter receptor genes (Glycine R, glutamate R, serotonin R, dopamine R), insulin-like-peptide, and one of the junction protein genes (lachesin) in the gut and brain of naïve vs. antibiotic-treated mosquitoes (Figure 6b). Consistent with NTs quantitative data, the respective receptor genes also showed a significant difference in their abundance throughout the gut-brain axis. We also noticed a differential expression pattern of ILP3, ARMAA (aromatic-L-amino-acid decarboxylase/serotonin synthesizing enzyme), and lachesin transcript between naïve and antibiotic-treated mosquitoes undergoing a metabolic switch event (Figure 6b). With our current data, we propose that a bi-directional microbiome–gut–brain axis communication may exist to manage complex gut immune-physiological responses in gravid females.

## 4. Discussion

Host-seeking and blood-feeding behavior evolution makes it difficult to resolve the complexity of decision-making neuro-actions in hematophagous insects [3]. Recently, Benjamin J. Matthews et al. cataloged hundreds of genes that are differentially expressed in the blood-fed brain [54], of which the brain-encoded neuropeptide Y has been suggested to play a crucial role in host-seeking suppression following blood-feeding [4]. However, an in-depth analysis of the gut-metabolic-switching and the modulation of brain function is unexplored. Our study attempts to establish a molecular relationship of the gut–brain–axis (GBA) communication, and explore the possible functional correlation of gut-endosymbionts on neuro-transmitters dynamics influencing GBA communication.

### 4.1. Gut-Metabolic Switch Modulates the Brain’s Energy Metabolism and Functional Engagement

To understand the effect of blood meal in modulating brain functions, we performed a comparative RNA-Seq analysis of naïve sugar-fed and blood-fed adult female mosquitoes’ brains in *An. culicifacies*. Blood-feeding induces an exclusive induction of oxidation-reduction family genes and a comparative pathway analysis predicts that blood meal may enhance the brain’s energy metabolic activities. In contrast to the pre-blood meal olfactory responses, which are significantly influenced by age/sex/circadian rhythm [1,27,28], we did not observe any significant alteration of the neuro-modulator genes expression in the brain of the non-blood-fed aging female mosquitoes. The existing literature suggests that the onset of host-seeking behavior in mosquitoes coincides with the age-dependent change in the relative abundance of olfactory genes and an increase in the sensitivity of the olfactory sensory neurons (OSNs) [47,48,49]. Therefore, we interpret and correlate that the appearance of oxidation-reduction category genes following blood-meal is a unique feature of the mosquito brain, independent of aging, possibly to regulate metabolic switch activities.

Shreds of evidence from *Drosophila*, vertebrates, and limited studies in mosquitoes also suggest that altered metabolic physiology influences the cross-talk between the brain and peripheral tissues for the maintenance of systemic energy homeostasis [11,33,55,56,57,58]. To perform this action, continuous neuronal stimulation is required, which consequently increases the energy demand of the brain [57,59]. The enrichment of the fructose-mannose metabolic pathway and the persistent elevation of PGC-1 and oxoglutarate dehydrogenase gene provide evidence of escalated energy metabolism and enhanced mitochondrial activity in blood-fed mosquitoes’ brains [29,30]. In this context, it is plausible to propose that enhanced mitochondrial activity may increase the ROS level, which could have a deleterious impact on the neuro actions. Our observation of the unique appearance of the pentose phosphate pathway and glutathione peroxidase transcripts (oxidation-reduction category gene), along with the upregulation of CLIP-domain serine proteases and peroxidases immune transcripts (Figure S7a,b) may attribute to the scavenging of ROS generated due to enhanced mitochondrial activity. Moreover, the blood-meal-induced expression of amino acid transporters and trehalose transporter indicated that both trehalose and amino acids may serve as a raw material for enhanced energy metabolism. Furthermore, we also observed a significant alteration of transcripts involved in intracellular signaling than neurotransmitter receptors in the blood-fed mosquitoes’ brains. Taken together, we hypothesize that an internal nutritional stimulus may shift the brain’s engagement from external communication to inter-organ management, which requires a rapid and continuous synaptic transmission, neurotransmitter recycling, and axo-dendritic transport, resulting in enhanced energy metabolism in the brain [57,58].

### 4.2. Neuromodulatory Responses Establish Brain-Distant Organ Communication

To support our hypothesis, we profiled a selected class of neuromodulators, neuropeptides, and neurohormones genes expression in the brain and correlated their impact on distant organs. Corresponding to the innate physiological status, we observed a time-dependent change in the expression pattern of the respective transcripts in the brain, and other targeted tissues of mosquitoes such as midgut, malpighian tubule, and ovaries. But, in turn, how these neuromodulatory responses reinforce brain action remains unknown. Recent studies in *Drosophila* suggest that leukokinin neuropeptide regulates protein diet-induced post-prandial sleep and minimized movement [60]. We also observed a transient increase in leukokinin, and its receptor gene in the brain, and sustained up-regulation of the leukokinin receptor gene in the gut till 30 h of blood-feeding. These data support the idea that until the blood meal gets digested in the gut, the brain may undergo ‘food coma’ and restrict external communication, but may actively engage in managing inter-organ communications (through ILPs and other neuro-hormones e.g., DH44, OEH, etc.). Compared to the brain, significant and sustained modulation of neuro-modulators in the gut of blood-fed mosquitoes even after decapitation, further suggested a specialized ability of the gut to serve as a “second brain” possibly to share and minimize the function of the primary brain [15]. Taken together, we interpret that gut-metabolic-switching may favor the establishment of a bidirectional ‘gut-brain axis communication in the gravid female mosquitoes, though the detailed molecular mechanism is yet to unravel.

### 4.3. Neurotransmitter Signaling and Microbiome Alteration Influences Gut-Brain-Axis Communications

Neurotransmitters, including both biogenic amines and amino acids, are well-known endogenous chemicals, that influence rapid inter-organ signal transmission and decision-making abilities [61,62]. To clarify and establish a possible functional correlation between the gut metabolic switch and gut-brain axis communication, we quantified the levels of neurotransmitters secreted from both gut and brain tissues. When compared to the naïve sugar-fed status, an unusual and paramount shift from the brain to the gut was observed for almost all the neurotransmitter levels after blood feeding. An enhancement of aspartic acid, histidine, tryptophan, GABA, and histamine levels in blood-fed mosquitoes’ gut may be a consequence of the rapid degradation of protein-rich blood meal in the mosquito gut [63]. Although the effect of tyrosine enrichment in the brain is intriguing, however, an undetectable level of tyrosine in the gut (Figure S4a) supports previous findings that the scavenging of toxic tyrosine from the gut is essential for the safeguarding journey of blood-fed mosquitoes [64]. A substantial body of literature also suggests that biogenic amines such as dopamine and serotonin are the critical regulators of feeding, host-seeking, and cognitive functions [65,66,67,68,69]. Thus, it would be worth testing whether an increase in the precursor molecules of dopamine i.e., tyrosine, in the blood-fed mosquito’s brain (Figure S4b), improves the cognitive power of the mosquitoes following the first blood-meal exposure. Likewise, an enrichment of tryptophan, a precursor of serotonin, may favor the minimization of the host-seeking behavioral activities of gravid females (Figure S4) [70]. Additionally, ~25-fold upregulation of GABAergic neurotransmission upon blood-feeding in the midgut further highlights its possible function in the regulation of innate immune response by activating the autophagy due to gut flora expansion (Figure S4a) [71,72].

Appreciably, a recent term ‘psycobiotics’, which aims to examine the influential effect of the microbiome on the gut–brain axis communication, is common to vertebrate’s neurobiology, but insects’ communities are the least attended [73,74]. In vertebrates, studies suggested that mediators of the microbiota–gut–brain–axis communication are usually affected by microbial metabolism which includes short-chain fatty acids such as butyrate, neurotransmitters e.g., serotonin and γ-aminobutyric acid (GABA), hormones e.g., cortisol, and other immune system modulators e.g., quinolinic acid [75]. Further research on vertebrates and fruit flies indicated that gut microbiota influences several behavioral physiologies, including host metabolism, appetite, mood, sensory perception, and cognition [76,77,78,79,80]. Recent studies in flies also demonstrated that gut commensal bacteria and the composition of dietary amino acid supplements greatly influence in shaping the behavioral responses such as food choice and olfactory-guided foraging decisions [81,82,83]. However, studies on mosquitoes’ gut-symbionts are predominantly limited to their impact on parasite growth and their potentiality for para-transgenic approaches [81].

Our observation of a rapid proliferation of *Pseudomonas bacterial* sp. in the gut of blood-fed mosquitoes may likely be due to increased consumption of dietary tryptophan for the synthesis of serotonin, correlating a possible cause for the suppression of appetite (Figure 3c and Figure 4) [82,83]. Additionally, a significant reduction of the excitatory neurotransmitters glutamic acid and aspartic acid in the brain may help to restrict the foraging behavior in gravid females [84]. However, a parallel 10-fold increase in aspartic acid in the gut is whether a result of gut-microbial metabolism, and/or any correlation with gut-neuro-endocrine regulation for egg development remains uncertain. Previous biochemical characterization of Locust’s vitellogenin protein showed that it carries high content of aspartic acid, glutamic acid, and leucine [85]. An independent in-silico amino-acid composition analysis of *An. culicifacies* vitellogenin protein (AEO51020.1) also revealed a high content of aspartic acid (6.2%), glutamic acid (6.7%), phenylalanine (7.6%), and serine (8.7%). Furthermore, previous literature indicated that disruption of gut-microbiota by antibiotic treatment not only reduces the anti-*Plasmodium* immunity but also hinders the egg development in the blood-feeding mosquitoes [20,86]. Therefore, we correlate that blood-meal-induced gut-microbial metabolism and activation of the vitellogenesis process may sequester a substantial amount of amino acids to nurture the eggs [45]. But, the remaining major fraction of amino acids either serves as an energy reservoir in the fat body [45] or functions as a neurotransmitter, possibly to maintain gut-brain-axis communication, though further studies are needed to prove these presumptions.

Additionally, emerging evidences claimed that histamine, the potent NTs, may influence endosymbionts-host interactions and modulate host immune responses. Histamine sensing and chemotaxis properties are observed in *Pseudomonas* sp. and *E. coli*. Simultaneously, *Enterobacteriaceae* family members are found to secrete histamine. Though the influential effect of histamine signaling on the gut-microbiome dynamics in mosquitoes has not been explored yet, our observation of 100-fold enrichment of histamine with consequent proliferation (~40%) of *Psedomonodales* family members in blood-fed mosquitoes’ gut indicates the putative role of histamine on endosymbionts proliferation. To enlighten this hypothesis, we performed the knock-down study with the histamine-gated-chloride-channel gene and profiled the total microbial population as well as immune genes in the gut of naïve-sugar-fed and blood-fed mosquitoes. Significant modulation of gut-microbial proliferation and upregulation of the immune genes (cecropin2, cecropin3 and defensin1) in the blood-fed knock-down mosquitoes indicating that histamine may not only manage gut-metabolic switch activity, but also influence gut endosymbionts proliferation and dynamics, though elucidating these signaling mechanism requires future efforts.

A noteworthy modulation of gut neurotransmitters encourages us to test whether the blood-meal-induced rapid proliferation of gut flora influences gut-brain axis communications. We disrupted the gut endosymbionts by providing an antibiotic diet orally supplement to the newly emerged mosquitoes for 4–5 days and observed a ~0.2–40-fold difference in the abundance of neurotransmitters in both the gut and the brain. Surprisingly, we also noticed aggressive feeding behavior of aseptic adult female mosquitoes towards the vertebrate host (personal observation). An earlier study showed that germ-free mice exhibited stress-induced altered behavioral response, which was restored after complete microbiota recolonization [87]. Studies further signify that the microbial antigens such as lipopolysaccharide (LPS) and lipoteichoic acids generated in response to antibiotic treatment elicit immune responses, and favors early development of the gut-brain axis communication via gut neuronal sensing [88]. In the mosquito *An. stephensi*, the antibiotic treatment also enhances the transcriptional responses of gut-immune peptides, but how it affects neuro-sensing remains unclarified [89]. We interpret that a higher abundance of histamine in the brain and GABA in the gut of antibiotic-treated mosquitoes may be accountable for the enhanced host-seeking behavioral activities, either directly through neuro-stimulation or indirectly through the vagal pathway [90,91]. Furthermore, blood-feeding to aseptic mosquitoes resulted in a multi-fold up-regulation of serine and glutamic acid suggesting a limited usage of the respective amino acids, in the lack of a microbial population (Figure S7 and Table S5), which consumes crucial amino acids to synthesize the building blocks of bacterial cell wall components in the healthy blood-fed mosquitoes [92,93].

## 5. Conclusions

The current investigation provides a novel insight into how gut–metabolic–switch-induced transcriptional modulation shifts mosquito’s brain engagement from external communication (pre-blood meal host-seeking and host selection) to inter-organ communication (post-blood meal physiological homeostasis) for the fitness of the mosquitoes. Although evidence is available on the physiological effects of the gut microbiota on whole-body function in health and disease, the role of gut-brain-axis communication is very limited in insects. To the best of our knowledge, our data provide initial evidence that correlates the potential role of gut endosymbionts in microbiome–gut–brain–axis communication in the mosquito. We believe our conceptual framework may be valuable to modify mosquitoes’ olfactory perception and cognition through the alteration of gut bacteria, and hence for new vector control tool development.

## Figures and Tables

**Table 1:** Details of the selected transcripts used to understand inter-organ communication during metabolic switch events.

Sl. No.	Gene Name	Synthesized from	Target Tissue	Possible Function	Target Tissue for Expression Study
1.	ILP1	MNSC of brain	Multiple tissues	Halt ovarian maturation [35]	Brain, midgut
2.	ILP3	MNSC of brain	Midgut, Ovary, Fat Body, Hemocyte	Nutrient storage by FB, regulation of digestive enzymes by MG, Ecdysteroid production from ovaries, the immune response by HC [8,36]	Brain, midgut
3.	Leucokinin	Abdominal ganglia	Gut, Malpighian tubule	Regulation of fluid secretion, ionic balance [36]	Brain
4.	PTTH—Prothoracicotropic Hormone	Brain	Not Known	Diapause and blood-feeding [37]	Brain
5.	Neuropeptide Y Receptor—NRY	NSC of brain	Brain	Host-seeking inhibition [4,38]	Brain
6.	Leucokinin Receptor	Multiple tissues	Multiple tissues	Regulation of fluid secretion, ionic balance [39]	Brain, midgut
7.	Diuretic hormone 44 (DH44)	Gut endocrine cells	Malpighian tubule	Regulation of diuresis [40]	Brain, midgut
8.	OEH—Ovary Ecdysteroidogenic Hormone	MNSC and ventricular ganglia of the brain	Ovary	Induces ecdysone production from the ovary after blood feeding [39]	Brain
9.	ARMAA—Aromatic-L-amino-acid decarboxylase	Multiple tissues	Multiple tissues	Synthesis of serotonin neurotransmitter and regulation of multiple physiological processes	Brain
10.	DH44R1	Malpighian tubule	Malpighian tubule	Regulation of Diuresis [39,40]	Midgut and Malpighian tubule
11.	CCHamide Receptor 2	CCHamide2 synthesized from gut endocrine cells	Multiple tissues	Nutrient dependent regulation of ILPs from brain [39]	Midgut
12.	5HTR—Serotonin Receptor	Multiple tissues	Multiple tissues	Multiple behavioral and physiological processes [41,42]	Brain, Midgut
13.	Glutamate R—Glutamate Receptor	Multiple tissues	Multiple tissues	Olfactory ionotropic glutamate receptor in odorant recognition (Identified from AC brain transcriptome data) [43]	Brain, Midgut
14.	Glycine R—Glycine Receptor	Multiple tissues	Multiple tissues	Inhibit neurotransmission (Identified from AC brain transcriptome data) [44]	Brain, Midgut
15.	Akt Kinase—Protein kinase B	Multiple	Multiple	Activation of TOR pathway [8]	Ovary
16.	CYP31A41-20E hydroxylase (20E synthesizing enzyme)	Ovary	Fat body and ovary	Ovary and oocyte development [45]	Ovary
17.	STPK—Serine threonine-protein kinase	Multiple	Multiple	Multiple physiological processes [46]	Brain, Ovary
18.	PI4-Kinase	Multiple	Multiple	Multiple physiological processes (Identified from AC brain transcriptome data)	Brain, Ovary
19.	Calcitonin Receptor	Malphigian tubule	Malphigian tubule	Regulation of diuresis [40,47]	Malphigian tubule
20.	KDNaCa Exchanger	Malpighian tubule	Malpighian tubule	Regulate fluid secretion and diuresis [40]	Malpighian tubule
21.	V-Type ATPase	Malpighian tubule	Malpighian tubule	Regulate membrane potential and diuresis [40]	Malpighian tubule

## Data Availability

The RNASeq sequencing data were deposited to National Centre for Biotechnology Information (NCBI) Sequence Reads Archive (SRA) system (BioProject accessions: PRJNA555826; BioSample accessions: SRR9853884 for Ac-Br-SF, SRR9853885 for Ac-Br-30 min, and SRR9853883 for Ac-Br-30 h). The metagenomics sequencing data were deposited to National Centre for Biotechnology Information (NCBI) Sequence Reads Archive (SRA) system including for Ac-MG-SF (SRR12579422), and for Ac-MG-BF (SRR12622557).

## Supplementary Materials

The following supporting information can be downloaded at: https://www.mdpi.com/article/10.3390/cells11111798/s1, Figure S1: A technical overview to decode the hard-wired genetic structure of brain system of *Anopheles culicifacies* mosquito; Figure S2: Differential gene expression analysis in aging non-blood fed female mosquito brain; Figure S3: Transcriptional Response of neuromodulator receptor genes in naïve and decapitated blood-fed female *An. culicifacies* mosquitoes; Figure S4: Fold change of NT abundance; Figure S5: Relative expression of *Agromonas* and *Rubrobacter;* Figure S6: Neurotransmitter’s dynamics of naïve and aseptic mosquitoes collected from sugar fed and blood fed conditions; Figure S7: Molecular catalog of brain-specific immune transcripts; Table S1: Validation DGE data; Table S2: List of Primer Sequence; Table S3: Annotation kinetics of RNA-Seq data Table S4: Comparative alpha diversity indices of metagenomics study; Table S5: Quantitative estimation of neurotransmitters and Representative UHPLC-MS/SRM chromatogram of Blank.

## References

[1] Das De T., Thomas T., Verma S., Singla D., Chauhan C., Srivastava V., Sharma P., Kumari S., Tevatiya S., Rani J. (2018). A synergistic transcriptional regulation of olfactory genes drives blood-feeding associated complex behavioral responses in the mosquito anopheles culicifacies. Front. Physiol..

[2] Potter C.J. (2014). Stop the biting: Targeting a mosquito’s sense of smell. Cell.

[3] Das De T., Dixit R. (2020). Neuro-Olfactory Regulation and Salivary Actions: A Coordinated Event for Successful Blood-Feeding Behavior of Mosquitoes. Sino-Nasal and Olfactory System Disorders.

[4] Duvall L.B., Ramos-Espiritu L., Barsoum K.E., Glickman J.F., Vosshall L.B. (2019). Small-Molecule Agonists of Ae. aegypti Neuropeptide Y Receptor Block Mosquito Biting. Cell.

[5] Beyenbach K., Petzel D. (2017). Diuresis in Mosquitoes: Role of a Natriuretic Factor. Physiology.

[6] Beyenbach K.W. (2012). A dynamic paracellular pathway serves diuresis in mosquito Malpighian tubules. Ann. N. Y. Acad. Sci..

[7] Sanders H.R., Evans A.M., Ross L.S., Gill S.S. (2003). Blood meal induces global changes in midgut gene expression in the disease vector, *Aedes aegypti*. Insect Biochem. Mol. Biol..

[8] Badisco L., Van Wielendaele P.V., Broeck J. (2013). Vanden Eat to reproduce: A key role for the insulin signaling pathway in adult insects. Front. Physiol..

[9] Liu Q., Jin L.H. (2017). Organ-to-Organ Communication: A Drosophila Gastrointestinal Tract Perspective. Front. Cell Dev. Biol..

[10] Duvall L.B. (2019). Mosquito Host-Seeking Regulation: Targets for Behavioral Control. Trends Parasitol..

[11] Droujinine I.A., Perrimon N. (2016). Interorgan Communication Pathways in Physiology: Focus on *Drosophila*. Annu. Rev. Genet..

[12] Gulia-Nuss M., Robertson A.E., Brown M.R., Strand M.R. (2011). Insulin-like peptides and the target of rapamycin pathway coordinately regulate blood digestion and egg maturation in the mosquito *Aedes aegypti*. PLoS ONE.

[13] Fadda M., Hasakiogullari I., Temmerman L., Beets I., Zels S., Schoofs L. (2019). Regulation of Feeding and Metabolism by Neuropeptide F and Short Neuropeptide F in Invertebrates. Front. Endocrinol..

[14] Christ P., Reifenrath A., Kahnt J., Hauser F., Hill S.R., Schachtner J., Ignell R. (2017). Feeding-induced changes in allatostatin-A and short neuropeptide F in the antennal lobes affect odor-mediated host seeking in the yellow fever mosquito, *Aedes aegypti*. PLoS ONE.

[15] Mayer E.A. (2011). Gut feelings: The emerging biology of gut-"brain communication. Nat. Rev. Neurosci..

[16] Ridaura V., Belkaid Y. (2015). Gut microbiota: The link to your second brain. Cell.

[17] Fülling C., Dinan T.G., Cryan J.F. (2019). Gut Microbe to Brain Signaling: What Happens in Vagus. Neuron.

[18] Forsythe P., Kunze W.A. (2013). Voices from within: Gut microbes and the CNS. Cell. Mol. Life Sci..

[19] Lampe L., Jentzsch M., Kierszniowska S., Levashina E.A. (2019). Metabolic balancing by miR-276 shapes the mosquito reproductive cycle and Plasmodium falciparum development. Nat. Commun..

[20] Romoli O., Gendrin M. (2018). The tripartite interactions between the mosquito, its microbiota and Plasmodium. Parasites Vectors.

[21] Sharma P., Sharma S., Mishra A.K., Thomas T., Das De T., Rohilla S.L., Singh N., Pandey K.C., Valecha N., Dixit R. (2015). Unraveling dual feeding associated molecular complexity of salivary glands in the mosquito *Anopheles culicifacies*. Biol. Open.

[22] Livak K.J., Schmittgen T.D. (2001). Analysis of relative gene expression data using real-time quantitative PCR and the 2(-Delta Delta C(T)) Method. Methods.

[23] Natarajan N., Ramakrishnan P., Lakshmanan V., Palakodeti D., Rangiah K. (2015). A quantitative metabolomics peek into planarian regeneration. Analyst.

[24] Ramesh D., Brockmann A. (2019). Mass Spectrometric Quantification of Arousal Associated Neurochemical Changes in Single Honey Bee Brains and Brain Regions. ACS Chem. Neurosci..

[25] Sharma P., Rani J., Chauhan C., Kumari S., Tevatiya S., Das De T., Savargaonkar D., Pandey K.C., Dixit R. (2020). Altered Gut Microbiota and Immunity Defines Plasmodium vivax Survival in Anopheles stephensi. Front. Immunol..

[26] Götz S., García-Gómez J.M., Terol J., Williams T.D., Nagaraj S.H., Nueda M.J., Robles M., Talón M., Dopazo J., Conesa A. (2008). High-throughput functional annotation and data mining with the Blast2GO suite. Nucleic Acids Res..

[27] De T.D., Sharma P., Rawal C., Kumari S., Tavetiya S., Yadav J., Hasija Y., Dixit R. (2017). Sex specific molecular responses of quick-to-court protein in Indian malarial vector *Anopheles culicifacies*: Conflict of mating versus blood feeding behaviour. Heliyon.

[28] Das De T., Hasija Y., Dixit R. (2018). Transcriptional responses of attractin gene in the mosquito Anopheles culicifacies: A synergistic neuro-olfactory regulation. J. Vector Borne Dis..

[29] Austin S., St-Pierre J. (2012). PGC1 and mitochondrial metabolism—Emerging concepts and relevance in ageing and neurodegenerative disorders. J. Cell Sci..

[30] Lin J., Handschin C., Spiegelman B.M. (2005). Metabolic control through the PGC-1 family of transcription coactivators. Cell Metab..

[31] Liang H., Ward W.F. (2006). PGC-1α: A key regulator of energy metabolism. Adv. Physiol. Educ..

[32] Shukla E., Thorat L.J., Nath B.B., Gaikwad S.M. (2015). Insect trehalase: Physiological significance and potential applications. Glycobiology.

[33] Mattila J., Hietakangas V. (2017). Regulation of carbohydrate energy metabolism in Drosophila melanogaster. Genetics.

[34] Hou Y., Wang X.L., Saha T.T., Roy S., Zhao B., Raikhel A.S., Zou Z. (2015). Temporal Coordination of Carbohydrate Metabolism during Mosquito Reproduction. PLoS Genet..

[35] Sim C., Denlinger D.L. (2009). A shut-down in expression of an insulin-like peptide, ILP-1, halts ovarian maturation during the overwintering diapause of the mosquito *Culex pipiens*. Insect Mol. Biol..

[36] Kersch C.N., Pietrantonio P.V. (2011). Mosquito Aedes aegypti (L.) leucokinin receptor is critical for in vivo fluid excretion post blood feeding. FEBS Lett..

[37] Zhang Q., Denlinger D.L. (2011). Molecular structure of the prothoracicotropic hormone gene in the northern house mosquito, Culex pipiens, and its expression analysis in association with diapause and blood feeding. Insect Mol. Biol..

[38] Liesch J., Bellani L.L., Vosshall L.B. (2013). Functional and Genetic Characterization of Neuropeptide Y-Like Receptors in *Aedes aegypti*. PLoS Negl. Trop. Dis..

[39] Strand M.R., Brown M.R., Vogel K.J. (2016). Mosquito Peptide Hormones: Diversity, Production, and Function. Advances in Insect Physiology.

[40] Piermarini P.M., Esquivel C.J., Denton J.S. (2017). Malpighian tubules as novel targets for mosquito control. Int. J. Environ. Res. Public Health.

[41] Kinney M.P., Panting N.D., Clark T.M. (2014). Modulation of appetite and feeding behavior of the larval mosquito Aedes aegypti by the serotonin-selective reuptake inhibitor paroxetine: Shifts between distinct feeding modes and the influence of feeding status. J. Exp. Biol..

[42] Ling L., Raikhel A.S. (2018). Serotonin signaling regulates insulin-like peptides for growth, reproduction, and metabolism in the disease vector *Aedes aegypti*. Proc. Natl. Acad. Sci. USA.

[43] Chen Q., Man Y., Li J., Pei D., Wu W. (2017). Olfactory ionotropic receptors in mosquito aedes albopictus (Diptera: *Culicidae*). J. Med. Entomol..

[44] Bowery N.G., Smart T.G. (2006). GABA and glycine as neurotransmitters: A brief history. Br. J. Pharmacol..

[45] Hansen I.A., Attardo G.M., Rodriguez S.D., Drake L.L. (2014). Four-way regulation of mosquito yolk protein precursor genes by juvenile hormone-, ecdysone-, nutrient-, and insulin-like peptide signaling pathways. Front. Physiol..

[46] Arsic D., Guerin P.M. (2008). Nutrient content of diet affects the signaling activity of the insulin/target of rapamycin/p70 S6 kinase pathway in the African malaria mosquito *Anopheles gambiae*. J. Insect Physiol..

[47] Coast G.M. (2005). Mosquito natriuretic peptide identified as a calcitonin-like diuretic hormone in *Anopheles gambiae* (Giles). J. Exp. Biol..

[48] Brown M.R., Clark K.D., Gulia M., Zhao Z., Garczynski S.F., Crim J.W., Suderman R.J., Strand M.R. (2008). An insulin-like peptide regulates egg maturation and metabolism in the mosquito *Aedes aegypti*. Proc. Natl. Acad. Sci. USA.

[49] Sharma A., Nuss A.B., Gulia-Nuss M. (2019). Insulin-like peptide signaling in mosquitoes: The road behind and the road ahead. Front. Endocrinol..

[50] Vogel K.J., Brown M.R., Strand M.R. (2015). Ovary ecdysteroidogenic hormone requires a receptor tyrosine kinase to activate egg formation in the mosquito *Aedes aegypti*. Proc. Natl. Acad. Sci. USA.

[51] Solari P., Rivelli N., De Rose F., Picciau L., Murru L., Stoffolano J.G., Liscia A. (2017). Opposite effects of 5-HT/AKH and octopamine on the crop contractions in adult Drosophila melanogaster: Evidence of a double brain-gut serotonergic circuitry. PLoS ONE.

[52] Guégan M., Zouache K., Démichel C., Minard G., Tran Van V., Potier P., Mavingui P., Valiente Moro C. (2018). The mosquito holobiont: Fresh insight into mosquito-microbiota interactions. Microbiome.

[53] O’Mahony S.M., Clarke G., Borre Y.E., Dinan T.G., Cryan J.F. (2015). Serotonin, tryptophan metabolism and the brain-gut-microbiome axis. Behav. Brain Res..

[54] Matthews B.J., McBride C.S., DeGennaro M., Despo O., Vosshall L.B. (2016). The neurotranscriptome of the *Aedes aegypti* mosquito. BMC Genom..

[55] Plaçais P.Y., De Tredern É., Scheunemann L., Trannoy S., Goguel V., Han K.A., Isabel G., Preat T. (2017). Upregulated energy metabolism in the Drosophila mushroom body is the trigger for long-term memory. Nat. Commun..

[56] Oriach C.S., Robertson R.C., Stanton C., Cryan J.F., Dinan T.G. (2016). Food for thought: The role of nutrition in the microbiota-gut-brain axis. Clin. Nutr. Exp..

[57] Yellen G. (2018). Fueling thought: Management of glycolysis and oxidative phosphorylation in neuronal metabolism. J. Cell Biol..

[58] Roh E., Song D.K., Kim M.S. (2016). Emerging role of the brain in the homeostatic regulation of energy and glucose metabolism. Exp. Mol. Med..

[59] Volkenhoff A., Weiler A., Letzel M., Stehling M., Klämbt C., Schirmeier S. (2015). Glial glycolysis is essential for neuronal survival in drosophila. Cell Metab..

[60] Murphy K.R., Deshpande S.A., Yurgel M.E., Quinn J.P., Weissbach J.L., Keene A.C., Dawson-Scully K., Huber R., Tomchik S.M., Ja W.W. (2016). Postprandial sleep mechanics in Drosophila. eLife.

[61] Mittal R., Debs L.H., Patel A.P., Nguyen D., Patel K., O’Connor G., Grati M., Mittal J., Yan D., Eshraghi A.A. (2017). Neurotransmitters: The Critical Modulators Regulating Gut–Brain Axis. J. Cell. Physiol..

[62] Holzer P., Farzi A. (2014). Neuropeptides and the microbiota- Gut-brain axis. Advances in Experimental Medicine and Biology.

[63] Imamura T., Baldwin T.O., Riggs A. (1972). The amino acid sequence of the monomeric hemoglobin component from the bloodworm, Glyat liver. J. Biol. Chem..

[64] Sterkel M., Perdomo H.D., Guizzo M.G., Barletta A.B.F., Nunes R.D., Dias F.A., Sorgine M.H.F., Oliveira P.L. (2016). Tyrosine Detoxification Is an Essential Trait in the Life History of Blood-Feeding Arthropods. Curr. Biol..

[65] French A.S., Simcock K.L., Rolke D., Gartside S.E., Blenau W., Wright G.A. (2014). The role of serotonin in feeding and gut contractions in the honeybee. J. Insect Physiol..

[66] Kamhi J.F., Arganda S., Moreau C.S., Traniello J.F.A. (2017). Origins of Aminergic Regulation of Behavior in Complex Insect Social Systems. Front. Syst. Neurosci..

[67] Friedman D.A., Pilko A., Skowronska-Krawczyk D., Krasinska K., Parker J.W., Hirsh J., Gordon D.M. (2018). The Role of Dopamine in the Collective Regulation of Foraging in Harvester Ants. iScience.

[68] Vinauger C., Lahondère C., Wolff G.H., Locke L.T., Liaw J.E., Parrish J.Z., Akbari O.S., Dickinson M.H., Riffell J.A. (2018). Modulation of Host Learning in Aedes aegypti Mosquitoes. Curr. Biol..

[69] Vinauger C. (2019). Vector cognition and neurobiology. Curr. Opin. Insect Sci..

[70] Ngai M., Shoue D.A., Loh Z., McDowell M.A. (2019). The pharmacological and functional characterization of the serotonergic system in Anopheles gambiae and Aedes aegypti: Influences on flight and blood-feeding behavior. Sci. Rep..

[71] Kim J.K., Kim Y.S., Lee H.M., Jin H.S., Neupane C., Kim S., Lee S.H., Min J.J., Sasai M., Jeong J.H. (2018). GABAergic signaling linked to autophagy enhances host protection against intracellular bacterial infections. Nat. Commun..

[72] Zhu Y., Zhang R., Zhang B., Zhao T., Wang P., Liang G., Cheng G. (2017). Blood meal acquisition enhances arbovirus replication in mosquitoes through activation of the GABAergic system. Nat. Commun..

[73] Sarkar A., Lehto S.M., Harty S., Dinan T.G., Cryan J.F., Burnet P.W.J. (2016). Psychobiotics and the Manipulation of Bacteria–Gut–Brain Signals. Trends Neurosci..

[74] Montiel-Castro A.J., González-Cervantes R.M., Bravo-Ruiseco G., Pacheco-López G. (2013). The microbiota-gut-brain axis: Neurobehavioral correlates, health and sociality. Front. Integr. Neurosci..

[75] Parker A., Fonseca S., Carding S.R. (2020). Gut microbes and metabolites as modulators of blood-brain barrier integrity and brain health. Gut Microbes.

[76] van de Wouw M., Schellekens H., Dinan T.G., Cryan J.F. (2017). Microbiota-Gut-Brain Axis: Modulator of Host Metabolism and Appetite. J. Nutr..

[77] Kawase T., Nagasawa M., Ikeda H., Yasuo S., Koga Y., Furuse M. (2017). Gut microbiota of mice putatively modifies amino acid metabolism in the host brain. Br. J. Nutr..

[78] Westfall S., Lomis N., Prakash S. (2018). Longevity extension in Drosophila through gut-brain communication. Sci. Rep..

[79] Akami M., Andongma A.A., Zhengzhong C., Nan J., Khaeso K., Jurkevitch E., Niu C.Y., Yuval B. (2019). Intestinal bacteria modulate the foraging behavior of the oriental fruit fly *Bactrocera dorsalis* (Diptera: *Tephritidae*). PLoS ONE.

[80] Gareau M.G. (2014). Microbiota-gut-brain axis and cognitive function. Advances in Experimental Medicine and Biology.

[81] Blumberg B.J., Short S.M., Dimopoulos G. (2015). Employing the Mosquito Microflora for Disease Control. Genetic Control of Malaria and Dengue.

[82] Kaur H., Bose C., Mande S.S. (2019). Tryptophan Metabolism by Gut Microbiome and Gut-Brain-Axis: An in silico Analysis. Front. Neurosci..

[83] Jenkins T.A., Nguyen J.C.D., Polglaze K.E., Bertrand P.P. (2016). Influence of tryptophan and serotonin on mood and cognition with a possible role of the gut-brain axis. Nutrients.

[84] Taylor P., Brown J.H. (1943). Basic Neurochemistry: Molecular, Cellular and Medical Aspects. Basic Neurochemistry: Molecular, Cellular and Medical Aspects.

[85] Chen T.T., Strahlendorf P.W., Wyatt G.R. (1978). Vitellin and vitellogenin from locusts (*Locusta migratoria*). J. Biol. Chem..

[86] Gaio A.D.O., Gusmão D.S., Santos A.V., Berbert-Molina M.A., Pimenta P.F.P., Lemos F.J.A. (2011). Contribution of midgut bacteria to blood digestion and egg production in aedes aegypti (diptera: *Culicidae*) (L.). Parasites Vectors.

[87] Sudo N., Chida Y., Aiba Y., Sonoda J., Oyama N., Yu X.N., Kubo C., Koga Y. (2004). Postnatal microbial colonization programs the hypothalamic-pituitary-adrenal system for stress response in mice. J. Physiol..

[88] Mazzoli R., Pessione E. (2016). The neuro-endocrinological role of microbial glutamate and GABA signaling. Front. Microbiol..

[89] De T.D., Sharma P., Thomas T., Singla D., Tevatiya S., Kumari S., Chauhan C., Rani J., Srivastava V., Kaur R. (2018). Interorgan molecular communication strategies of “Local” and “Systemic” innate immune responses in mosquito Anopheles stephensi. Front. Immunol..

[90] Chen A., Singh C., Oikonomou G., Prober D.A. (2017). Genetic Analysis of Histamine Signaling in Larval Zebrafish Sleep. eNeuro.

[91] Bushey D., Tononi G., Cirelli C. (2015). Sleep- and wake-dependent changes in neuronal activity and reactivity demonstrated in fly neurons using in vivo calcium imaging. Proc. Natl. Acad. Sci. USA.

[92] Cava F., Lam H., De Pedro M.A., Waldor M.K. (2011). Emerging knowledge of regulatory roles of d-amino acids in bacteria. Cell. Mol. Life Sci..

[93] Aliashkevich A., Alvarez L., Cava F. (2018). New insights into the mechanisms and biological roles of D-amino acids in complex eco-systems. Front. Microbiol..

