# Supplementary material for: Bidirectional Microbiome-Gut-Brain-Axis Communication Influences Metabolic Switch-Associated Responses in the Mosquito Anopheles culicifacies
Source: Cells. 2022 May 31;11(11):1798. doi: 10.3390/cells11111798 (PMC9180301; doi:10.3390/cells11111798)

Supporting Information

Bidirectional Microbiome-Gut-Brain-Axis Communication Influences Metabolic Switch-associated Responses in the Mosquito *Anopheles culicifacies*

Tanwee Das De [1,2]† , Punita Sharma[1]†, Sanjay Tevatiya[1], Charu Chauhan[1], Seena Kumari[1], Pooja Yadav, Deepak Singla[1,2], Vartika Srivastava[1], Jyoti Rani[1], Yasha Hasija[3], Kailash C Pandey[1,] and Rajnikant Dixit [1*].

[1]Laboratory of Host-Parasite Interaction Studies, National Institute of Malaria Research, Sector-8, Dwarka, Delhi 110077, India.
[2] Department of Biology, Indian Institute of Science Education and Research, Dr. Homi Bhabha Road, Pashan, Pune 411008, Maharashtra, India
[3] School of Agricultural Biotechnology, Punjab Agricultural University, Ludhiana, Punjab 141027, India
4 Department of Biotechnology, Delhi Technological University. Shahbad Daulatpur, Main Bawana Road, Delhi 110042, India;

*****Correspondence: dixitrk@mrcindia.org
†These authors contributed equally to this work.

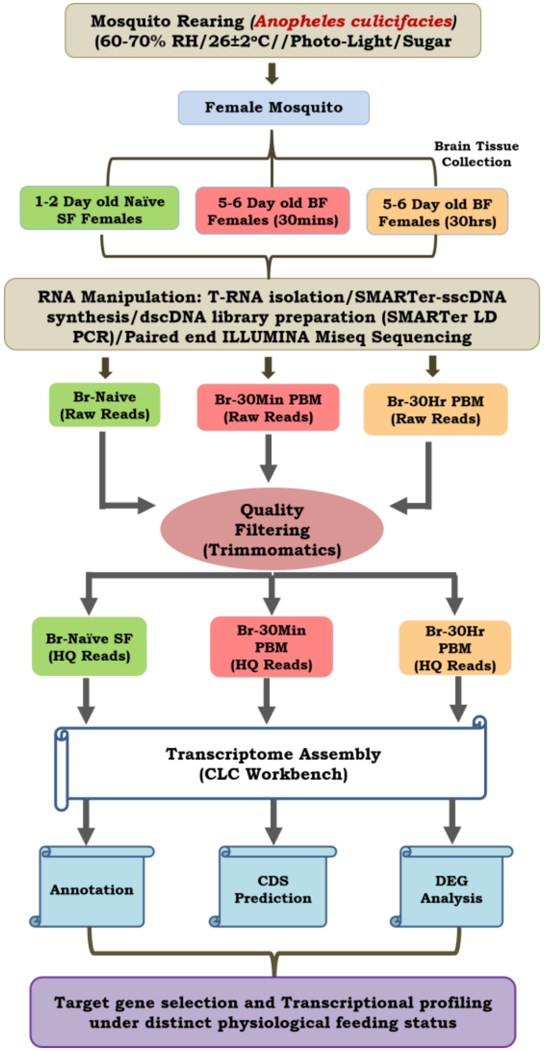

Figure S1. A technical overview to decode the hard-wired genetic structure of brain system of *Anopheles culicifacies* mosquito.

Table S1. Validation DGE data.

Gene Name	Transcriptome data/DEG	Real Time Validation
serine/threonine-protein kinase (STPK)	Br_30hr = -1.2-Fold Down<br>Br-30min = - 5.7-Fold Down	10hr = Upregulation<br>30min and 30hr = Downregulation
Neurotransmitter gated ion channel (NTGIC)	No Change	No Change
Glycine receptor (Gly Receptor)	No Change	No Change
Gamma-aminobutyric acid type B receptor (GABA Receptor)	Br_30hr = -1.1-fold down<br>Br_30min = -0.27-Fold Down	10hr = Non-significant Upregulation<br>30min and 30hr = Downregulation
Glutamate receptor (Glut Receptor)	No Change	No Change
phosphatidylinositol-Phosphilipase C gamma (PLC)	Br-30hr = -0.2-Fold Down<br><br>Br-30min = -0.13-Fold Down	10hr = Upregulation<br>30min and 30hr = Downregulation

Table S2. List of Primer Sequence used in the study.

Sl. No.	Primer Name and Sequence
**1.**	Actin_Fw: 5' TGCGTGACATCAAGGAGAAG 3'<br>Actin_Rev: 5' GATTCCATACCCAGGAACGA 3'
**2.**	Ac_PyruvateKinase_Fw: 5’ CGCACTTGATCTCCAAGTAT 3’<br>Ac_PyruvateKinase_Rev: 5’ TTCCAGCCAGTAACAACAA 3’
**3.**	Ac_Solute Carrier 7_Fw: 5’ TCAATAGCTCCGAAATCAGT 3’<br>Ac_Solute Carrier 7_Rev: 5’ TGATAACGAACAGCAAGACA 3’
**4.**	Ac_AATransporter_Fw: 5’ CAATGCCTATGGTTACAGGT 3’<br>Ac_AATransporter_Rev: 5’ GCTGGTAAGTGTCCTTCTTG 3’
**5.**	Ac_TrehalaseTransporter_Fw: 5’ CGATGGGACTGTACTTCTTC 3’<br>Ac_TrahalaseTransporter_Rev: 5’ GTCTAGATCGGCGAAAAAC 3’
**6.**	Ac_PGC1_Fw: 5’ ACCTTACGGTAAAATCGTCA 3’<br>Ac_PGC1_Rev: 5’ GTACGGTAGCTGATGTTCGT 3’
**7.**	Ac_OxoglutarateDHS_Fw: 5’ GCAACTACTTCCATCTGCTC 3’<br>Ac_OxoglutarateDHS_Rev: 5’ GAGCCTTCAACAAGTCGTAA 3’
**8.**	cGMP PK_Fw: 5’ GCGTTTGATTATCTGCACTC 3’<br>cGMP PK_Rev: 5’ AAGGACTCCAAGTGACCAGT 3’

**9.**	GlutamateR_Fw: 5'AGTGGTATCAACGCAGAGTG 3'<br><br>GlutamateR_Rev: 5' GAGTTTAAGCACTGCTCCAC 3'
**10.**	Glycine R_Fw: 5' GATACTGCCACTACCTCGTC 3'<br><br>Glycine R_Rev: 5' CTTGGAGACCGAATTGAATC 3'
**11.**	GABA R_Fw: 5' CAGAACGAAGAAGGCTACTC 3'<br><br>GABA R_Fw: 5' AGTATCCACGCATACTCAGC 3'
**12.**	ARMAA_Decarboxylase_Fw: 5'GGTAACCAAGTCCTTCAGTG 3'<br><br>ARMAA_Decarboxylase_Rev: 5' TAGAACAGACGACCTCGAAC 3'
**13.**	ILP1_Fw: TCCACTACATGGAAAACTCC<br><br>ILP1_Rev: GTCATCAGTGCCTGGTAGAT
**14.**	ILP3_Fw: TAGCAATGATGAGTGGATGA<br><br>ILP3_Rev: ACAACACTCCTCTACGATGC
**15.**	Leukokinin_Fw: AAACATCGCATAGCAGAGAT<br><br>Leukokinin_rev: TCAGATAATCCTGCACCATA
**16.**	NRY_Fw: TACTGTACGGCTGGTTGAAT<br><br>NRY_Rev: TTAGTTCCGGCAGTGTTC
**17.**	OEH_Fw: GACAAGAATGCGGTGATAAT<br><br>OEH_Rev: CGTTGCTGTAGTAATCGAAG
**18.**	DH44R1_Fw: CTCGAAATAGAATGCTCCTG<br><br>DH44R1_Rev: AGATGACGATGAGGTAGGTG

**19.**	LKR_Fw: AAGAGGGAACACGACAAAC<br><br>LKR_Rev: GCTCGATATAATTGGTGGTC
**20.**	DH44_Fw: AACGAACAGGAAGATCTCAA<br><br>DH44_Rev: ATACCGTAGACGTACCGTGA
**21.**	CCHAR2_Fw: CCACTCCGAAAACTACAGAC<br><br>CCHAR2_Rev: GTGGCAGGAAGTAGTAAACG
**22.**	V-Type ATPase_Fw: TTACATGTACACCGATTTGG<br><br>V-Type ATPase_Rev: GACTTCATCAGACGTGACAG
**23.**	DopR_Fw:5’ GTTATGGGCGTGTTTATTGT 3’<br><br>DopR_Rev:5’ GCTGGTACTTGCGTCTTATC 3’
**24.**	AKT Kinase_Fw: GATGGAGGAGGTAAAGTTCC<br><br>AKT Kinase_Rev: GAACTCACGGTCGAAGTAAC
**25.**	CYP314A1_Fw: GAGATTGCGCAAGAATTTAG<br><br>CYP314A1_Rev: GGAAGTTGTCCTCACTCTGA
**26.**	PTTH_Fw: CTTCACCTCTGAATTGCTTC<br><br>PTTH_Rev: ACAAGAAGACGGGTACTGTG
**27.**	KDNaCaExchanger_Fw: GTGAGATGGGTATCAGCAAC<br><br>KDNaCaExchanger_Rev: CTTCCAATCAAGTTTGAAGC

**28.**	GABA ClCh_Fw: 5’ GGAAGGTGTTTGGTAAGTCA 3’<br><br>GABA ClCh_Rev: 5’ GGTGATCGTGTTCGAGTAAT 3’
**29.**	PLC_Fw: 5’ TGGATTCGTCCAACTATCAT 3’<br><br>PLC_Rev: 5’ TTCACGATCACCTCGTTC 3’
**30.**	PI-4Kinase_Fw: 5’ ACATCATCTCCTCACTGTCC 3’<br><br>PI-4Kinase_Rev: 5’GTGTGCCACTGTTGTAATCA 3’
**31.**	ST ProteinKinase_Fw: 5’TTTATAGTGCCGTGTGTTGA 3’<br><br>ST ProteinKinase_Rev: 5’CTTAATGTGGAACCGATCAT 3’
**32.**	Trehalase_Fw: 5’ GAAGAGGACAAACAGGACTA 3’<br><br>Trehalase_Rev: 5’ GTTCCGGTAACCATAGAAC 3’
**33.**	5-HT Receptor_Fw: 5’ ATGATCTCGCGTAACTCCTC 3’<br><br>5-HT Receptor_Rev: 5’ ATCGGATTGACCAGACTGC 3’
**34.**	TOR_Fw: GTAGAATGTTGGTGGTCGAT<br><br>TOR_Rev: ACCATCTGCTAGGTTATTGC
**35.**	Octopamine Receptor_Fw: 5’CTACTGGCGGATCTATCGGG 3’<br><br>Octopamine Receptor_Rev: 5’ TGGTGGAAGGCTGTGTTTTG 3’
**36.**	Calcitonin R_Fw: AATAGAATGCTGGATGAACG<br><br>Calcitonin R_Rev: GGACGAAACGGTGTAAGTAT

**37.**	NTGated IonCh_Fw: 5’ ACGTTTCGAAAGTCAAACAC 3’<br><br>NTGated IonCh_Rev: 5’ GCTGTAGAATGCACAAATGA 3’

Table S3a. Annotation kinetics of RNA-Seq data.

**Molecular Features**	**Ac-Br-Naive**	**Ac-Br-30M PBM**	**Ac-Br-30Hr PBM**
**Total No. of Raw Reads**	5268211	3947521	3760078
**Total No. of Contigs**	32118	32984	38512
**Total Transcripts**	9460	9146	7387
**Total BLASTx hits (NR)**	8,668 (~91%)	8,336 (~91%)	6,548 (~88%)
**Transcripts with GO Match**			
**Molecular Function**	4773	4556	3575
**Biological process**	4446	4299	3381
**Cellular component**	2523	2424	1888

Table S3b. Percentage of differentially expressed transcripts.

Sample	No. of Transcripts	Transcripts showing Differential gene Expression (DGE)	Upregulated Transcripts	Downregulated Transcripts	Percentage of Transcripts showing DGE
Ac_Br_naive vs Ac_Br_30min	(9460 + 9146) = 18606	Total - 4747<br>Significant – 3183<br>Not significant - 1564	622 (3%)	2110 (11%)	14% CDS show differential expression
Ac_Br_Naive vs Ac_Br_30hr	(9460 + 7387) = 16847	Total -3966<br>Significant – 3174<br>Not significant - 792	482 (2%)	2469 (14%)	16 % show differential expression

Table S4. Comparative alpha diversity indices estimation of gut-bacterial population of naïve sugar fed and blood fed mosquito *An. culicifacies*.

Sample	Taxonomy Rank: Class	
	**Shannon index (H)/(h/LN(N))**	**Simpson index**
**Ac_SF_MG**	3.233/0.6802	0.9081
**Ac_BF_MG**	2.815/0.6424	0.888

Table S5. Quantitative estimation of 13 different neurotransmitters in the brain and the gut of mosquitoes under different physiological conditions.

Name of NT	Control SF_Br	Ab+_SF BR	Control SF_MG	Ab+_SF _MG	10hr PBM_Br	Ab+_10hr PBM_Br	10hr PBM_MG	Ab+_10hr PBM_MG	24hr PBM_Br	Ab+_24hr PBM_Br	24hr PBM_MG	Ab+_24hr PBM_MG
**Histidine**	607.77	622.52	356.52	469.70	397.58	1562.75	DC	3672.30	550.28	908.02	1968.73	DC
**Serine**	182.75	176.07	167.62	134.24	182.04	318.04	798.51	2636.08	197.18	350.75	1034.20	2830.31
**Histamine**	16.08	76.89	3.35	3.83	48.28	82.21	108.12	83.16	73.12	50.42	136.69	284.97
**Aspartic Acid**	272.35	96.74	115.69	24.02	209.89	143.05	1205.17	146.59	102.76	222.80	1312.08	2348.14
**Glutamic Acid**	604.78	255.68	513.97	397.82	523.25	425.24	DC	4530.44	486.92	690.13	DC	DC
**GABA**	300.01	240.89	6.38	14.50	174.40	300.76	153.31	72.01	175.86	321.81	126.79	13.94
**Dopa**	1.06	1.94	DC	2.00	1.45	2.04	DC	DC	2.08	1.84	DC	DC
**Octopamine**	5.49	0.39	BLQ	BLQ	2.29	0.20	NF	DC	0.65	1.11	NF	NF
**Tyrosine**	84.26	58.49	96.41	53.93	140.86	117.51	DC	DC	147.28	136.63	DC	136.63
**Dopamine**	1.12	0.28	0.13	0.46	1.00	0.62	69.17	26.02	1.06	0.83	11.59	25.18
**Serotonin**	9.33	2.25	1.92	1.35	6.06	3.39	143.62	81.14	3.59	3.72	32.70	81.37
**Tyramine**	0.08	BLQ	0.05	0.06	0.10	0.05	4.06	1.73	0.07	0.05	0.90	0.22
**Tryptophan**	21.84	68.84	11.59	16.44	36.93	74.66	1230.41	1054.85	41.40	40.70	2263.10	3650.53

*** BLQ= Below Limit of Quantitation**
NF= Not Found
DC= Detected but not calculated due to the highly suppressed Internal Standard signal

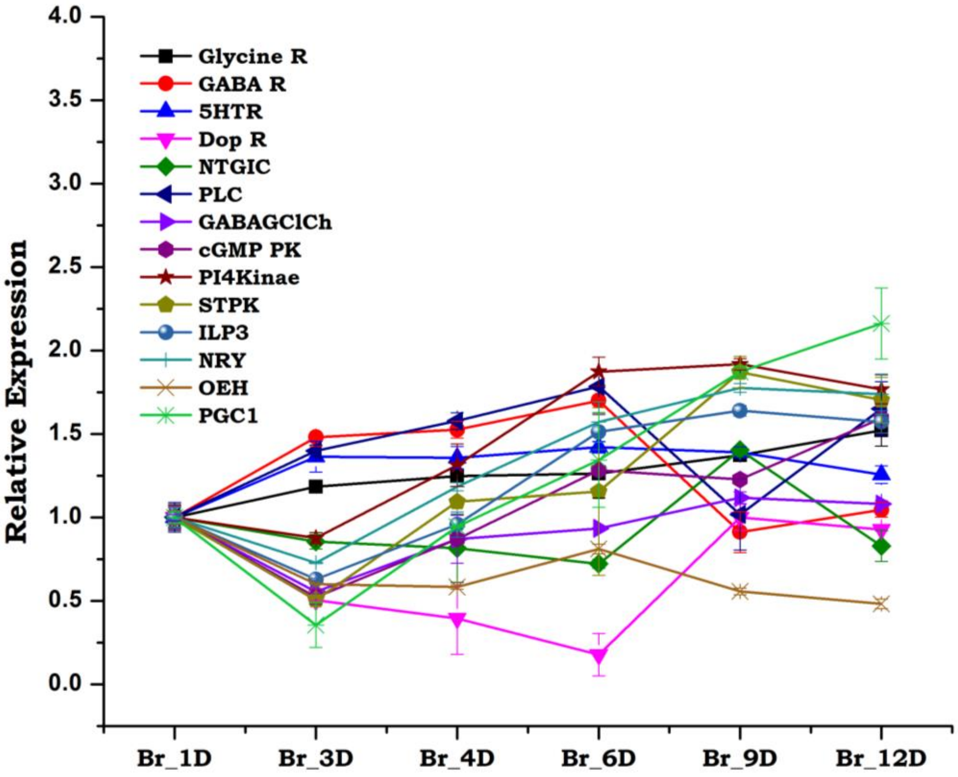

Figure S2. Differential gene expression analysis in aging non-blood fed female mosquito brain. No significant modulation of neuronal genes was observed when compared to 0-1-day old teneral mosquitoes with 12-day-old non-blood fed mosquitoes. Statistical analysis using two-way ANOVA followed by Tukey Test has implied at 0.05 level, the expression pattern of the respective genes was not statistically significant in aging mosquitoes *(n = 25, N = 3).*

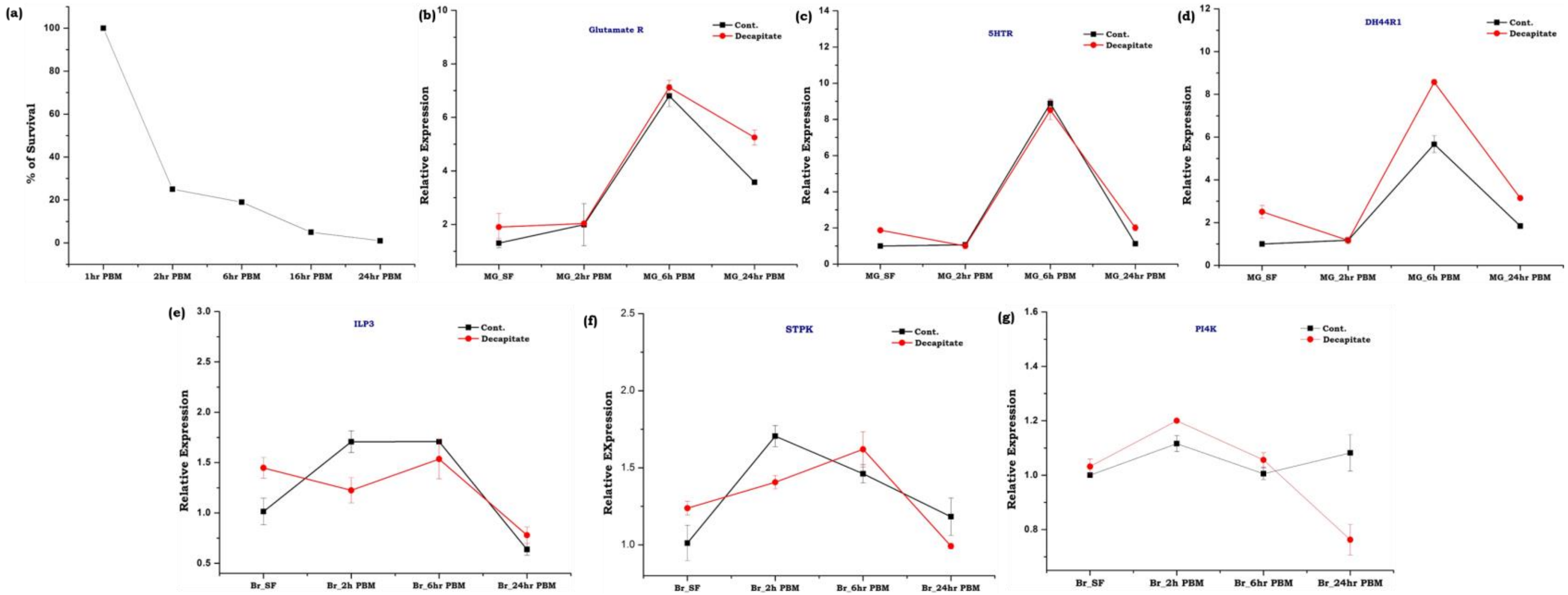

Figure S3. Transcriptional Response of neuromodulator receptor genes in naïve and decapitated blood-fed female *An. culicifacies* mosquitoes**.** (a) Percentage of mosquitoes that survived till 24 h after decapitation which was performed after 1h of blood-feeding. 5-6 days old sugar-fed mosquitoes were provided blood meal and mosquitoes heads were decapitated after 1 h. from the full-fed gravid females. After that, the decapitated mosquitoes were kept in a cage for recovery and count the live (mosquitoes that vibrate/move their legs or other body parts are considered as live) and dead mosquitoes (non-movable mosquitoes with visible shrinkage of the body parts at the respective time points are considered as dead) at different time points until we observed 100% mortality. The percentage of survival was calculated until 24h after blood feeding. (b-d) Relative gene expression analysis of neuromodulator receptor genes in the gut of blood-fed and decapitated female mosquitoes. (e-g) Relative gene expression analysis of neuromodulator receptor genes in the gut of blood-fed and decapitated female mosquitoes. Statistical analysis using two-way ANOVA implied that at 0.05 level the expression level the respective genes in control and decapitated females are not statistically different.

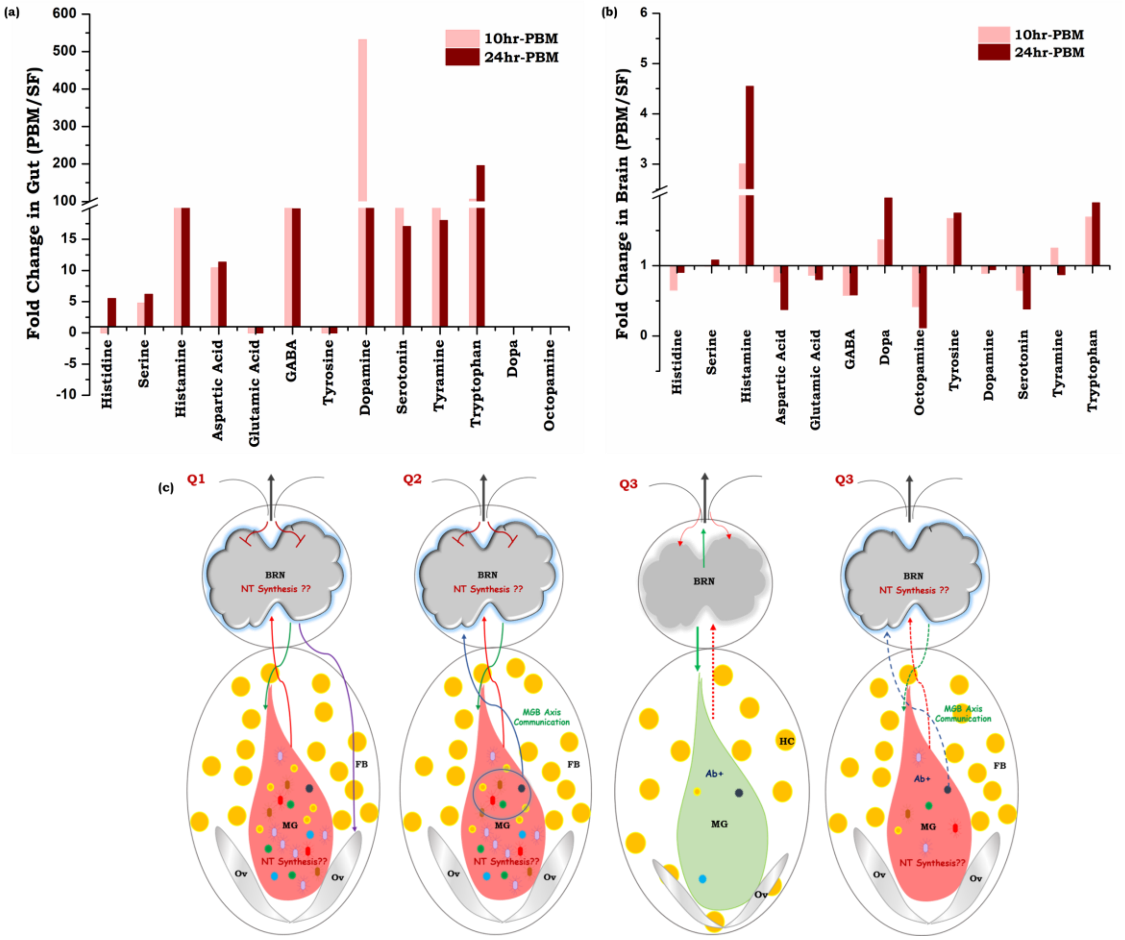

Figure S4. (a) Fold change of NT abundance in the gut of blood-fed mosquitoes. **(b)** Fold change of NT abundance in the brain of blood-fed mosquitoes. **(c)** Pictorial presentation demonstrating GBA communication in response to gut-metabolic switch in mosquitoes. Q1: Blood-feeding pauses external stimulus-guided neuro-olfactory responses, but may shift brain engagement through the vagus pathway (red arrow) to regulate actions in the distant organs such as the midgut (green arrow) and ovary (purple arrow). Here, we questioned whether increased levels of amino acids in the gut during blood meal digestion may act like an NT. Q2: Do blood-meal-induced gut flora proliferation (different colored shapes indicate diverse microbial flora) influence GBA communication in mosquitoes. Q3: Whether gut-bacterial removal by antibiotic treatment confers the establishment of microbiome-gut-brain axis (MGB) communication. BRN: Brain, MG: midgut, FB: Fat body, Ov: Ovary, Ab+: Antibiotic positive/treated.

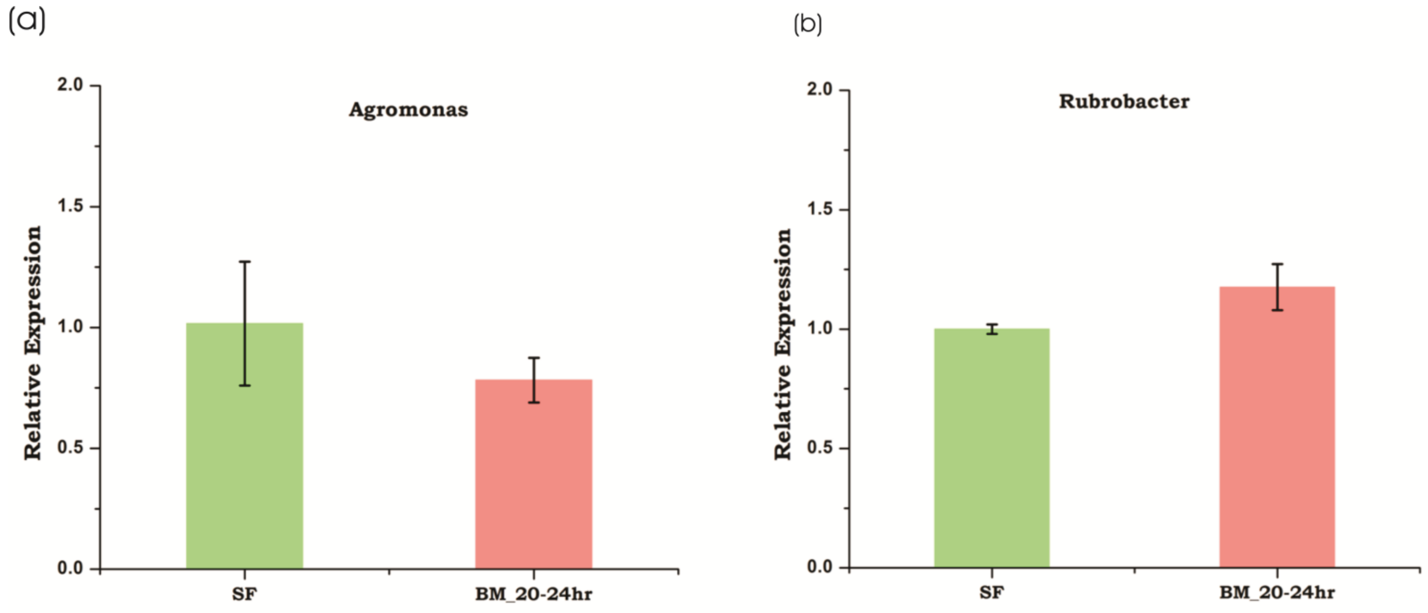

Figure S5. Relative expression of *Agromonas* and *Rubrobacter* (gram positive bacteria) at Sugar fed and 20-24hr Post blood fed condition shows no significant change during the two-feeding status.

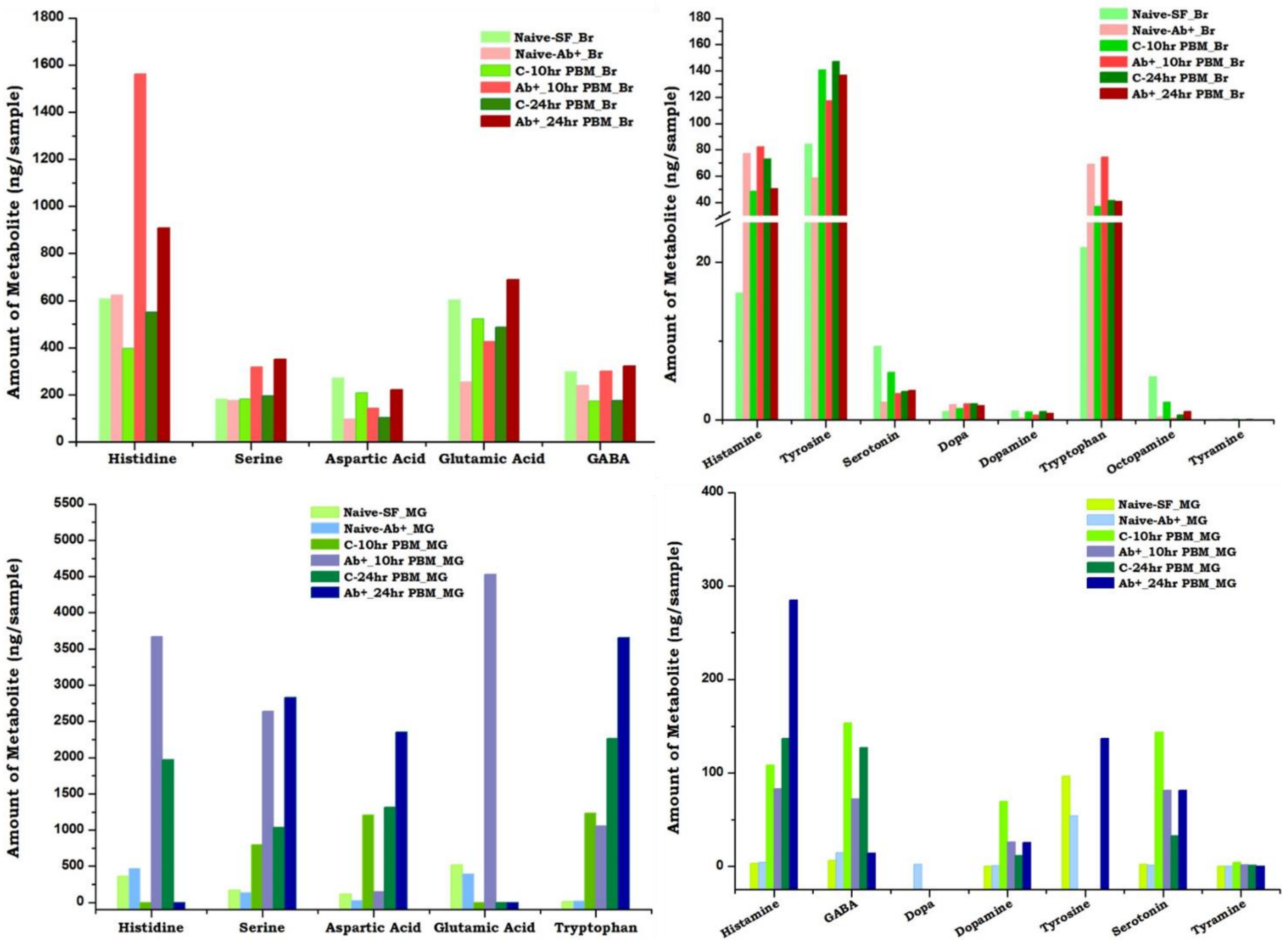

Figure S6. Neurotransmitter's dynamics of naïve and aseptic mosquitoes collected from sugar fed and blood fed conditions.

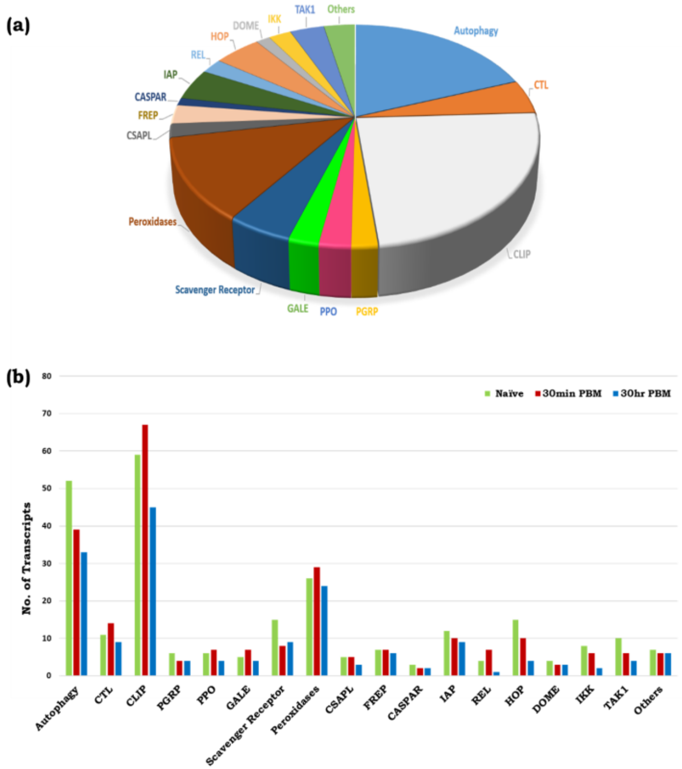

Figure S7. Molecular catalog of brain-specific immune transcripts. (a) Molecular catalog of the different classes of immune genes expressed in brain tissue; (b) Differential expression patterns of the brain immunome as determined by the number of sequences that appeared in each RNA-Seq data of naïve and blood-fed mosquito brains.

Representative UHPLC-MS/SRM chromatogram of Blank:

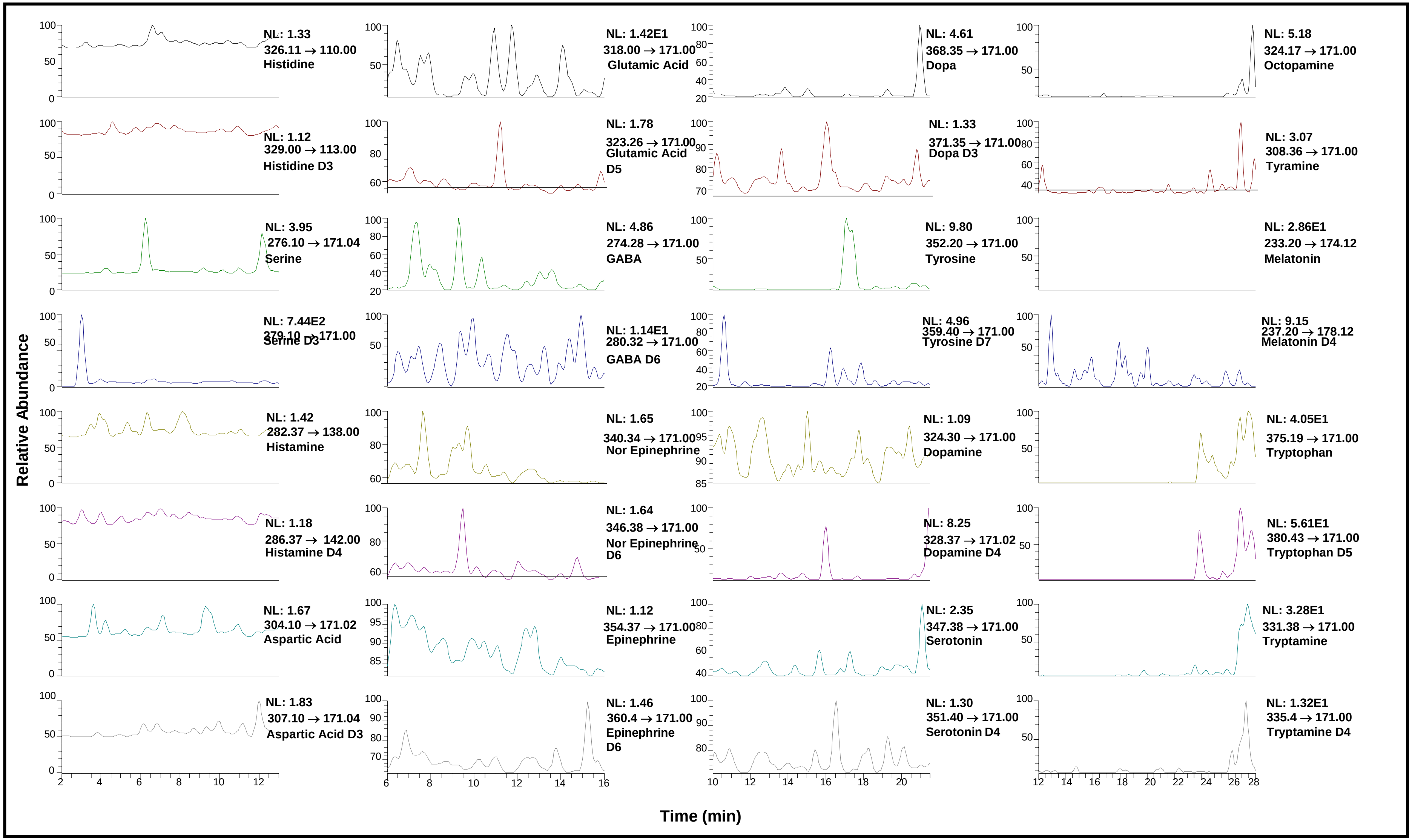

Representative UHPLC-MS/SRM chromatogram of Standard:

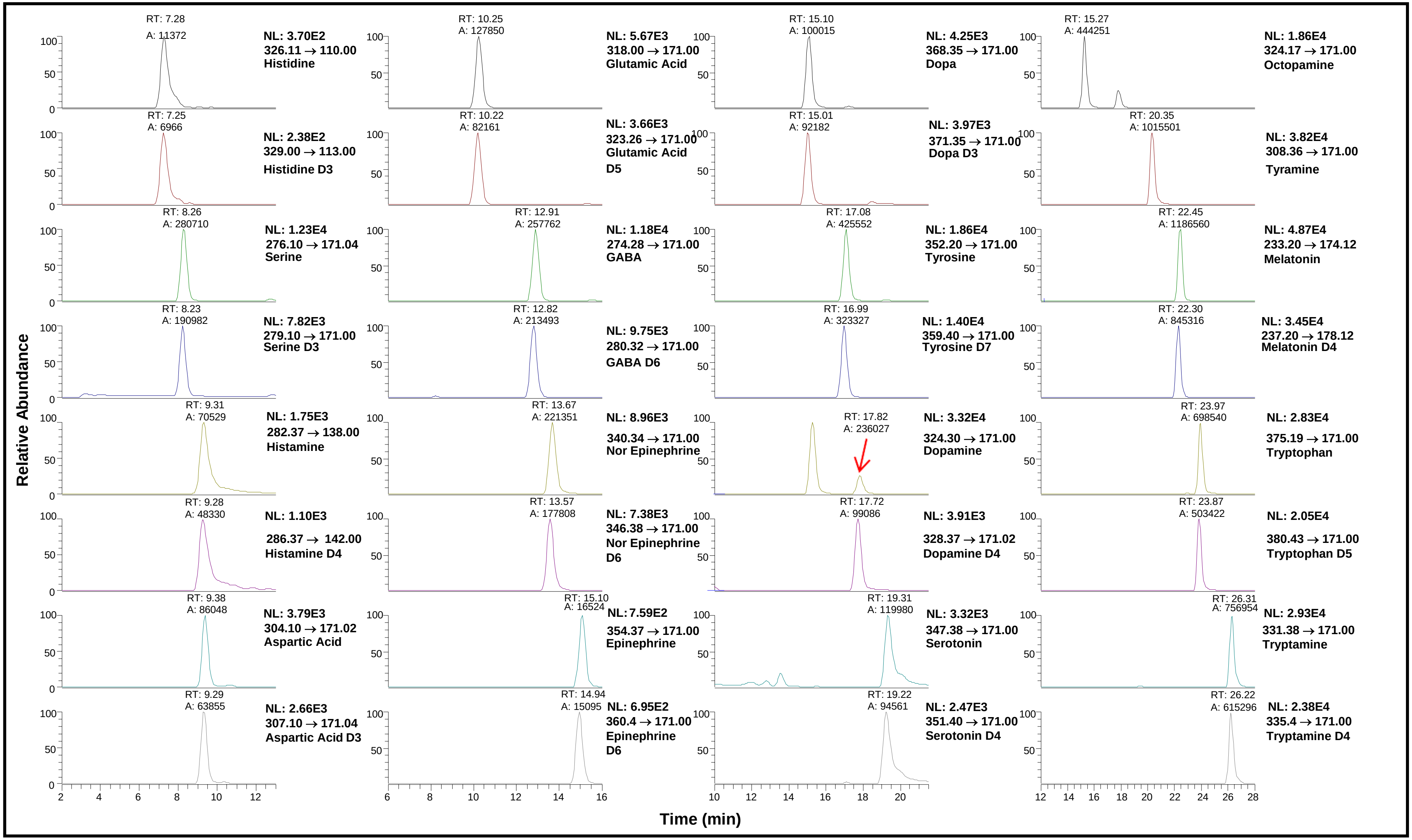

Representative UHPLC-MS/SRM chromatogram of sample Ac_SF_Br:

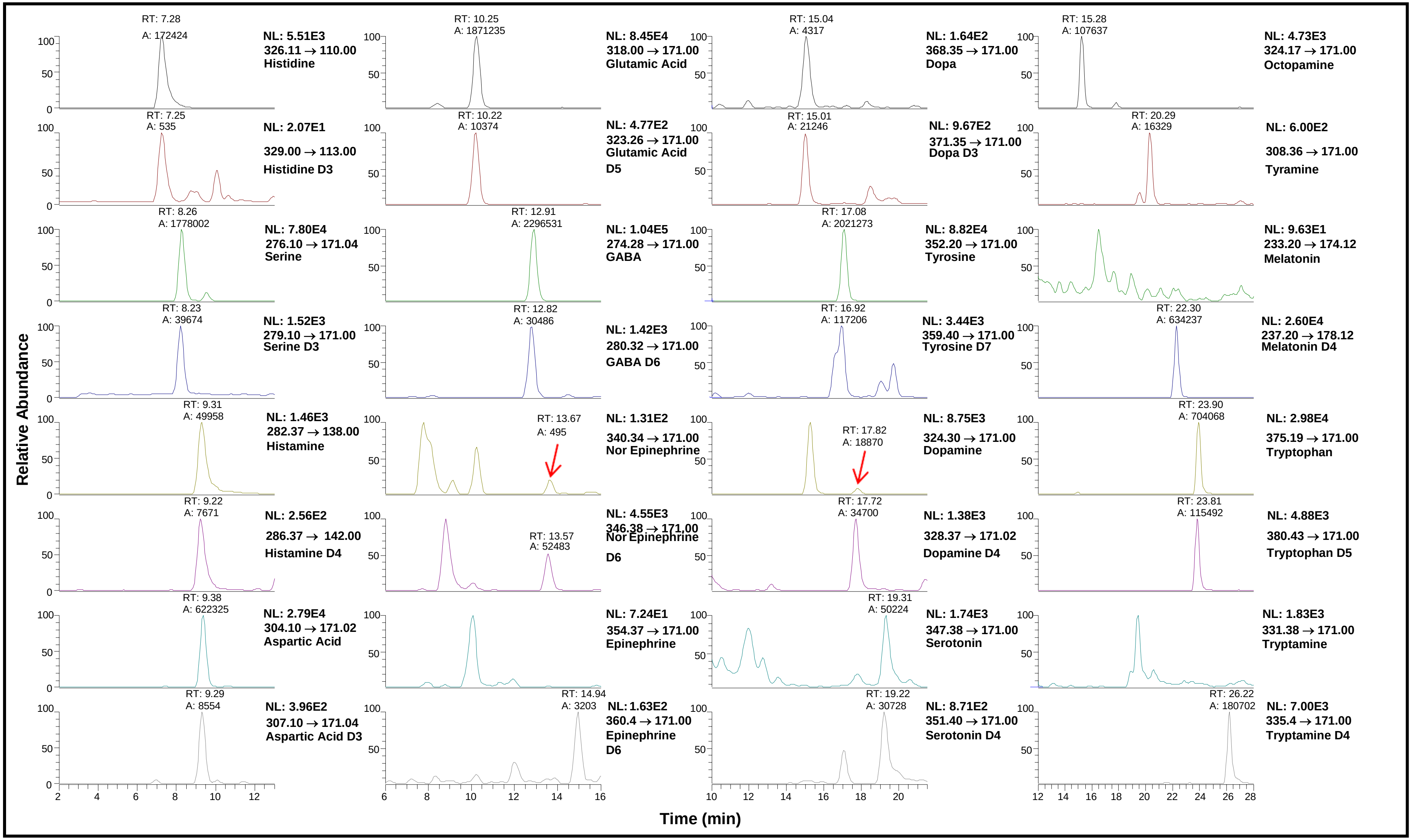

Representative UHPLC-MS/SRM chromatogram of sample Ac_SF_MG:

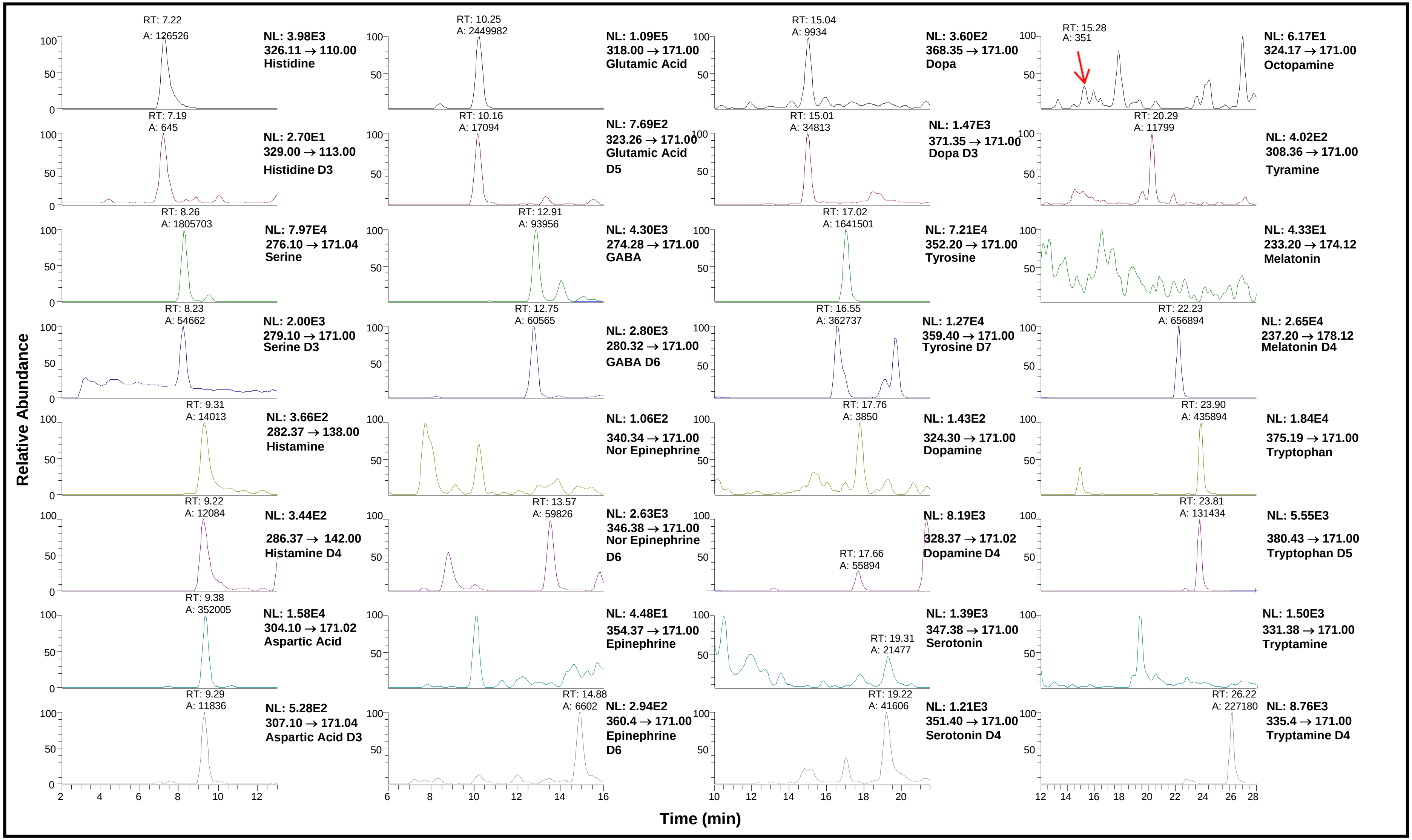

Supplement: Supplementary file 1 [file cells-11-01798-s001.zip › Supporting Information_Brain Paper.pdf]
